# Depletion of myeloid-derived Zbtb46^+^ cells improves glycemic control in obesity via the DPP4/GLP-1 pathway

**DOI:** 10.1016/j.jare.2025.10.032

**Published:** 2025-10-23

**Authors:** Shindy Soedono, Dan Hoang Nguyet Vo, Jiyeon Chang, Sharlene Sharlene, Princess Wendy Bayona, Sooyoung Kim, Jun Young Hong, Kae Won Cho

**Affiliations:** a Department of Integrated Biomedical Science, Soonchunhyang University, Cheonan 31151, the Republic of Korea; b Soonchunhyang Institute of Medi-Bio Science (SIMS), Soonchunhyang University, Cheonan 31151, the Republic of Korea; c Department of Systems Biology, College of Life Science and Biotechnology, Yonsei University, Seoul 03722, the Republic of Korea

**Keywords:** Obesity, Glucose intolerance, Insulin resistance, Adipose tissue, Dendritic cell, DPP4, GLP-1

## Abstract

**Introduction:**

Adipose tissue (AT) immune cells regulate metabolic functions in obesity through both inflammatory and non-inflammatory pathways. However, the specific roles and mechanisms of individual AT immune cell types in glycemic control remain poorly understood.

**Objective:**

This study investigates the function of myeloid-derived Zbtb46^+^ cells, a major subset of peripheral dendritic cells (DCs), in established obesity.

**Methods:**

Chimeric *Zbtb46*-DTR mice were generated by transplanting bone marrow from *Zbtb46*-DTR donors into wild-type recipients with distinct congenic markers. Obesity was induced with a high-fat diet (HFD; 60% kcal from fat), and myeloid-derived Zbtb46^+^ cells were selectively depleted in obese mice via diphtheria toxin (DT) injection. DC-specific *Dpp4* knockout (DC-*Dpp4*KO) mice were generated using the Cre-loxP system and subsequently challenged with the HFD.

**Results:**

Inducible depletion of myeloid-derived Zbtb46^+^ cells improves glucose homeostasis and reduces body weight in obese mice. Notably, these effects were observed even in weight-matched mice and under conditions of increased ATM accumulation, suggesting benefits independent of weight loss or AT inflammation. The improvement in glucose homeostasis was primarily mediated by elevated GLP-1 levels, which enhanced insulin secretion and decreased food intake. Increased GLP-1 was associated with decreased DPP4 activity, attributed to the depletion of ATDCs, a key contributor to circulating DPP4. Consistently, DC-specific *Dpp4* deficiency confirmed that ATDC-derived DPP4 regulates GLP-1–induced insulin secretion in obesity.

**Conclusions:**

These findings uncover a novel, non-inflammatory role for ATDCs in glucose regulation via the DPP4/GLP-1/GLP-1R axis, positioning them as promising therapeutic targets for obesity and related metabolic diseases.

## Highlights

• Dendritic cells (DCs) are key regulators of energy balance in established obesity.
• Inducible DC depletion in obesity enhances metabolic health, modulating glycemia through non-inflammatory mechanisms.
• Adipose tissue DCs (ATDCs) are critical regulators of DPP4 activity in obesity.
• ATDCs orchestrate the DPP4/GLP-1 axis, bridging immunity and metabolism.
• Targeting ATDCs presents a promising therapeutic strategy for obesity.

## Graphical abstract

**Figure:**
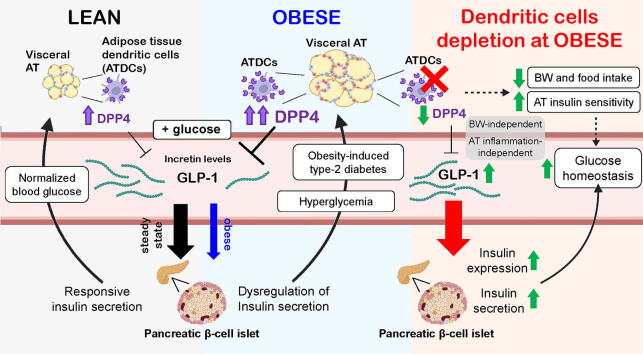
Adipose tissue dendritic cells (ATDCs) in visceral fat regulate glucose homeostasis in obesity through the DPP4/GLP-1/insulin axis.

## Introduction

Immune cells within adipose tissue (AT) are critical regulators of both local and systemic metabolic processes, maintaining homeostasis in health and significantly contributing to disease pathogenesis [[1], [2], [3], [4]]. In obesity, the immune landscape of AT undergoes substantial remodeling, shifting to a pro-inflammatory state characterized by increased secretion of cytokines and other inflammatory mediators [5]. This reconfiguration highlights the central role of immune cells in driving chronic AT inflammation, which contributes to AT dysfunction and disrupts systemic glucose and insulin metabolism [6]. Preclinical studies have shown that depleting pro-inflammatory immune cells or blocking immune cell infiltration improves glucose tolerance in obesity [7,8]. However, the outcomes of clinical interventions targeting inflammatory mediators have yielded only marginal benefits or limited efficacy in type 2 diabetes patients [9,10]. This discrepancy underscores the complexity of immune-mediated metabolic regulation and suggests the existence of non-inflammatory mechanisms by which immune cells regulate glucose homeostasis [4]. For instance, expression of glucagon-like peptide-1 receptor (GLP-1R) on colonic intraepithelial T cells has been shown to limit GLP-1 bioavailability, impairing glucose absorption and complicating glycemic regulation [11]. Additionally, retinoic acid production by islet-resident myeloid cells has been implicated in modulating insulin secretion and influencing glucose tolerance [12]. These findings suggest that the non-inflammatory functions of immune cells play a significant role in glucose homeostasis, highlighting the need for further investigation into their diverse roles in metabolic organs to find new therapeutic interventions for obesity-associated diseases.

Among the various immune cell populations in AT, ATDCs have emerged as novel, independent regulators of obesity-associated metabolic dysfunction. Previous studies have shown that ATDCs influence metabolism through the recruitment of ATMs and regulation of cytokine production [13,14]. As key intermediaries between innate and adaptive immunity, the MHC-II function of ATDC in obesity-related inflammation has also been characterized, promoting T helper (Th)-17 differentiation and suppressing regulatory T cell (Treg) populations [[13], [14], [15], [16]]. However, most studies examining ATDC function in obesity have relied on whole-body knockout mouse models, which are inadequate for defining and recapitulating their specific roles in obesity [17]. Mouse models targeting DC biogenesis, such as *Flt3l*^−/−^, *Csf2*^−/−^, and *Ccr7*^−/−^ mice, have encountered developmental challenges that may confound their effects on obesity progression [13,[18], [19], [20]]. Moreover, the shared CD11c expression between ATDCs and ATMs in obesity complicates role distinction in CD11c-Cre and CD11c-DTR mouse models [8,[21], [22], [23]]. Additionally, MHC-II knockout mice fail to differentiate the specific contributions of ATDCs and ATMs, given their overlapping roles as antigen-presenting cells [24,25]. These limitations obscure a precise understanding of ATDC function in established obesity, underscoring the need to elucidate their mechanisms in metabolic regulation *in vivo*.

Recently, non-immunomodulatory functions of ATDCs have also been identified, involving the expression of enzymes that regulate glucose, lipid, and amino acid metabolism [19,[26], [27], [28]]. For example, ATDCs modulate branched-chain amino acids (BCAA) and lysine metabolism in obesity, which correlates with enhanced peripheral insulin sensitivity [19]. Furthermore, ATDCs, alongside ATMs, have been implicated in the expression of DPP4, a key enzyme in glucose homeostasis that degrades incretin hormones and modulates insulin secretion [26,[29], [30], [31], [32]]. Despite these insights, current evidence remains largely descriptive and has yet to fully elucidate the specific role of ATDCs in the obese state, leaving their non-inflammatory contributions to metabolic regulation insufficiently understood. This raises critical questions about whether ATDCs predominantly exert inflammatory, non-inflammatory, or dual roles in obesity-related metabolic regulation.

Previously, we and other research groups have identified the exclusive expression of *Zbtb46* in ATDCs, providing a reliable marker to distinguish them from ATMs [8,33]. *Zbtb46* is a transcription factor specific to conventional DC (cDC) and their committed progenitors, while being absent in plasmacytoid DC (pDC), monocytes, macrophages, and other immune lineages [34,35]. The chimeric *Zbtb46*-DTR mouse model thus serves as a precise tool for observing peripheral DC functions, including those of ATDCs, while circumventing developmental and lethality issues associated with *Zbtb46* expression in endothelial cells and erythrocyte progenitors [17]. Here, using this inducible DC depletion model, we investigated the role of ATDCs in established obesity. Depleting myeloid-derived Zbtb46^+^ cells led to significant improvements in obesity-related outcomes and glycemic control. Notably, these improvements in glucose homeostasis occurred independently of both AT inflammation and weight reduction. Mechanistically, we identified a non-inflammatory role of ATDCs in regulating glucose homeostasis, primarily through DPP4/GLP-1/GLP-1R pathway. These findings uncover a previously unrecognized glucoregulatory function of ATDCs in obesity and highlight their potential as critical therapeutic targets for metabolic diseases.

## Material and methods

### Animal studies

CD45.1 C57BL/6J, B6.Cg-Tg(Itgax-cre)1-1Reiz/J (CD11c-Cre), and *Zbtb46*-DTR [B6(Cg)-*Zbtb46*^*tm1(HBEGF)Mnz*^/J); also referred to as zDC-DTR] were obtained from Jackson Laboratory. *Dpp4^tm1a(EUCOMM)Wtsi^* mice were obtained from the International Mouse Phenotyping Consortium (IMPC). To generate conditional knockout mice, the FRT-flanked selection cassette was excised, and animals were bred to homozygosity for the loxP-flanked *Dpp4* alleles (*Dpp4*^flox/flox^). These mice were subsequently crossed with CD11c-Cre transgenic mice to obtain CD11c-Cre; *Dpp4*^flox/flox^ mice (DC-*Dpp4*KO) and their Cre-negative littermate controls. Male mice were *ad libitum* fed either a regular chow as normal diet (ND; Pico Lab® Mouse Diet 20 #5058) or a 60% fat containing high fat diet (HFD; Research Diets #D12492). Diet-induced obesity (DIO) was induced by feeding mice a HFD for 12–16 weeks.

### Ethics statement

All experiments involving animals were conducted in accordance with the ethical guidelines and procedures approved by the Institutional Animal Care and Use Committee of Soonchunhyang University, Republic of Korea (Approval no: SCH20-0010). Human subject protocols were approved by the Institutional Review Boards of Soonchunhyang University Hospital (Approval no: SCHUH 2015-11-017), and written informed consent was obtained from all donors. The study was conducted in accordance with the principles outlined in the Declaration of Helsinki.

### Bone marrow transplantation (BMT) and myeloid-derived Zbtb46^+^ cell depletion

Bone marrow (BM) transplant cells were isolated from six-week-old donor mice by flushing BM cells from the femur and tibia with RPMI (Gibco) supplemented with 2% FBS (Gibco). The cells were filtered through 70 µm mesh, centrifuged at 550×*g* for 10 min at 4 ℃. Isolated BM cells were injected *retro*-orbitally into whole-body-irradiated (10 Gy) six-week-old CD45.1 recipient mice, 4 h after irradiation (10 million cells/mouse). For total depletion, BM cells from *Zbtb46*-DTR mice were used as the donor, while for partial depletion, BM cells from *Zbtb46*-DTR and CD45.1 mice were mixed in a 1:1 ratio. Reconstitution efficiency was confirmed to be >85% at six weeks post BM transplantation. The recipient mice were then fed either ND or HFD for 10–14 weeks to induce obesity.

Inducible depletion of myeloid-derived *Zbtb46*^+^ cells was achieved by intraperitoneal (i.p.) injection of diphtheria toxin (DT; Sigma-Aldrich) into chimeric *Zbtb46*-DTR mice every other day for 2 weeks while maintaining their respective diets (80 ng/g body weight for the first injection; 20 ng/g for subsequent injections). Control chimeric mice received equivalent injections of PBS. DT-mediated depletion selectively targets myeloid-derived Zbtb46^+^ cells, including pre-cDCs (immature cDCs) and conventional DC (cDC) subsets, and may also affect monocyte-derived DCs (moDCs) that exhibit phenotypic and functional overlap with cDCs under obese conditions. Plasmacytoid DCs (pDCs) were confirmed to remain unaffected.

### Metabolic measurement and blood samples

Body weights were measured weekly during HFD challenge and daily during the DT challenge. Glucose tolerance tests (GTT) were performed after a 16-hour fast by oral gavage of D-glucose (2 g/kg). Exendin-9 (Ex-9) (2 µg/g, i.p.) was injected 30 min prior to glucose loading, using a dose validated to impair glucose tolerance in HFD-fed but not ND-fed mice. Blood glucose concentrations (mg/dL) were measured at 0, 15, 30, 45, 60, 90, and 120 min after injection. Blood plasma was collected from the tail-vein at 0, 30, 60, 120 min post-injection. Plasma samples were immediately treated with a mixture of Diprotin A (Sigma; 0.1 mM), Aprotinin (Sigma; 5000KIU/mL), and EDTA (1.2 mg/mL), then centrifuged at 2000×*g* for 10 min and stored at −80 ℃. Insulin levels were measured using the Morinaga Ultra-Sensitive Mouse Insulin ELISA kit (MIOBS, #M1104). Plasma total GLP-1 was measured using the Mouse GLP-1 ELISA Kit (Crystal Chem, #81508) and plasma total GIP was measured using the Mouse GIP ELISA Kit (Crystal Chem, #81527). DPP4 levels were measured using the Mouse DPPIV/CD26 DuoSet ELISA (R&D #DY954). DPP4 activity in plasma was assayed using the DPP4 Activity Fluorometric Assay Kit (BioVision, #K779-100). The expression of metabolic hormones was analyzed in fasting and fed blood serum samples using the Luminex xMAP technology with the MILLIPLEX® Mouse Metabolic Hormone Expanded Panel (Merck Millipore, #MMHE-44K).

### Measurement of DPP4 activity from tissue

DPP4 tissue activity was measured using the DPP4 Activity Fluorometric Assay Kit (BioVision, #K779-100) following manufacturer’s protocol. Briefly, 10 mg of tissue from each mouse was homogenized in 4 volumes DPP4 assay buffer using a Dounce homogenizer while kept on ice, with 15 passes. The samples were then centrifuged for 10 min at 4 ℃ at 20,000×*g*. The supernatant was collected and used for the DPP4 activity assay, and results were normalized to protein concentration.

### Isolation of stromal vascular fraction from adipose tissue and flow cytometry

Stromal vascular cells (SVCs) were isolated from adipose tissue (AT) as previously described [36]. Briefly, excised AT was digested in digestion buffer containing 2 mg/mL type II collagenase for 25 min at 37 ℃, and then SVCs were separated by centrifugation. The cells were incubated with Fc Block for 10 min on ice and then stained with the indicated antibodies (Table S1) for 30 min at 4 °C. The stained cells were washed twice with PBS and fixed in 0.1% PFA before analysis. Flow cytometry analysis was performed using a BD FACS Canto II Flow Cytometer (BD Biosciences) and data were analyzed with FlowJo software (Treestar). Gating of ATDCs was performed from live CD45^+^ cells using CD64^−^ CD11c^+^ markers, which constitute the majority of cDCs. This strategy captures cDC1 (CD11b^−^ CD11c^+/hi^), cDC2 (CD11b^+^ CD11c^+^), and immature DC subsets (CD11b^−^ CD11c^+/mid^). The gating approach specifically excludes pDCs (CD11c^low^). Cell sorting for ATDCs (CD64^−^ CD11c^+^) and ATMs (CD64^+^) was performed using a BD FACS Aria Cell Sorting System. The sorted cells were seeded in complete RPMI medium supplemented with 10% FBS, 1% P/S, 1% MEM and 0.1% 2-mercaptoethanol.

### Immunohistochemistry and immunofluorescence

Human visceral AT (VAT) samples were obtained during bariatric surgery from patients at Soonchunhyang University Hospital, following the provision of written informed consent. Hematoxylin and eosin (H&E) staining was conducted on 5 µm-thick sections of AT paraffin tissue. Immunofluorescence (IF) was carried out on either paraffin tissue sections or whole AT, as described previously [16,24]. Pancreas samples were embedded in OCT compound and snap frozen. Pancreas tissue sections (10 µm-thick) were fixed in 4% PFA for 20 min, followed by permeabilization in PBS containing 0.3% Triton X-100 (Thermo). Sections were then blocked with 5 % BSA and incubated with the indicated antibodies (Table S1) overnight at 4 ℃, followed by incubation with secondary antibodies for 3 h at room temperature. DAPI (2 µg/mL) was used to stain the nuclei. Images were captured using a Leica-DMi8 Fluorescence Microscope and analyzed using ImageJ-win64 software. For GLP-1 immunofluorescence quantification, images were analyzed using MATLAB-based scripts. Tissue area, the number of GLP-1-positive cells, the total GLP-1-positive area, and fluorescence intensity were quantified for each section.

### Gene expression analysis

RNA was extracted from tissues using RiboEx™ (GeneAll) and cDNA was synthesized using the High-Capacity cDNA Reverse Transcription Kit (Applied Biosystems). SYBR Green Real-time PCR Master Mix (Toyobo) and QuantStudio1 (Applied Biosystems) were used for real-time quantitative PCR. *18s* expression was used as an internal control for data normalization. Samples were assayed in duplicate and relative expression was determined using the 2^−Δ^^Δ^CT method. Primers are listed in Table S2.

### Single-cell RNA sequencing (scRNA-seq) analysis

scRNA-seq datasets from human VAT were obtained from GEO (GSE176171) and the Single Cell Portal (SCP1903) and analyzed using Seurat v5.3.0. Seurat objects were generated with parameters min.cells = 3 and min.features = 200. Quality control filtering was applied as follows: for GSE176171, cells with *nFeature_RNA* < 7500, *nCount_RNA* < 50,000 were excluded; for SCP1903, cells with *nCount_RNA* < 40,000 or *percent.mt* < 10 were excluded. Only VAT samples were retained and stratified into lean (BMI 20–30) or obese (BMI > 40) groups. Datasets were merged, normalized using (*NormalizeData)*. Variable features were identified with FindVariableFeatures, followed by data scaling (*ScaleData*) and dimensionality reduction by principal component analysis (*RunPCA*). Integration across datasets was performed using the reciprocal PCA (RPCA) approach. The merged dataset was then used for neighbor graph construction (*FindNeighbors*), clustering (FindClusters), and visualization by uniform manifold approximation and projection (*RunUMAP*). For immune cell-focused analyses, clusters expressing *PTPRC* (CD45) were subsetted, re-processed using the same workflow (neighbor graph construction, clustering, UMAP visualization), and used for downstream analysis.

### Immunoblotting

For the insulin signaling assay, mice were fasted for 6 h and then stimulated with insulin (3 U/kg body weight) or saline via IP injection. Liver, muscle, and eWAT extracts were prepared 15 min after stimulation by homogenization using the Tissue Lyser II (Qiagen) in Pierce™ RIPA lysis buffer (Thermo Scientific) with a 1× protease inhibitor cocktail (BioRad). Protein concentrations were determined using the DC™ Protein Assay (BioRad). Proteins were separated by SDS-PAGE, then immunoblotted with the indicated antibodies (Table S1) overnight at 4 ℃ and visualized using Agfa CP1000.

### Statistical analysis

All values are expressed as means ± SEM. Statistical significance between two groups was assessed using an unpaired two-tailed Student's t*-*test, while differences among more than two groups were evaluated using one-way ANOVA. GraphPad Prism V8.0.2 was used for all statistical analyses. A *p-*value < 0.05 was considered statistically significant.

## Results

### Inducible depletion of myeloid-derived Zbtb46^+^ cells improves obesity-induced metabolic dysfunction

**Fig. 1:**
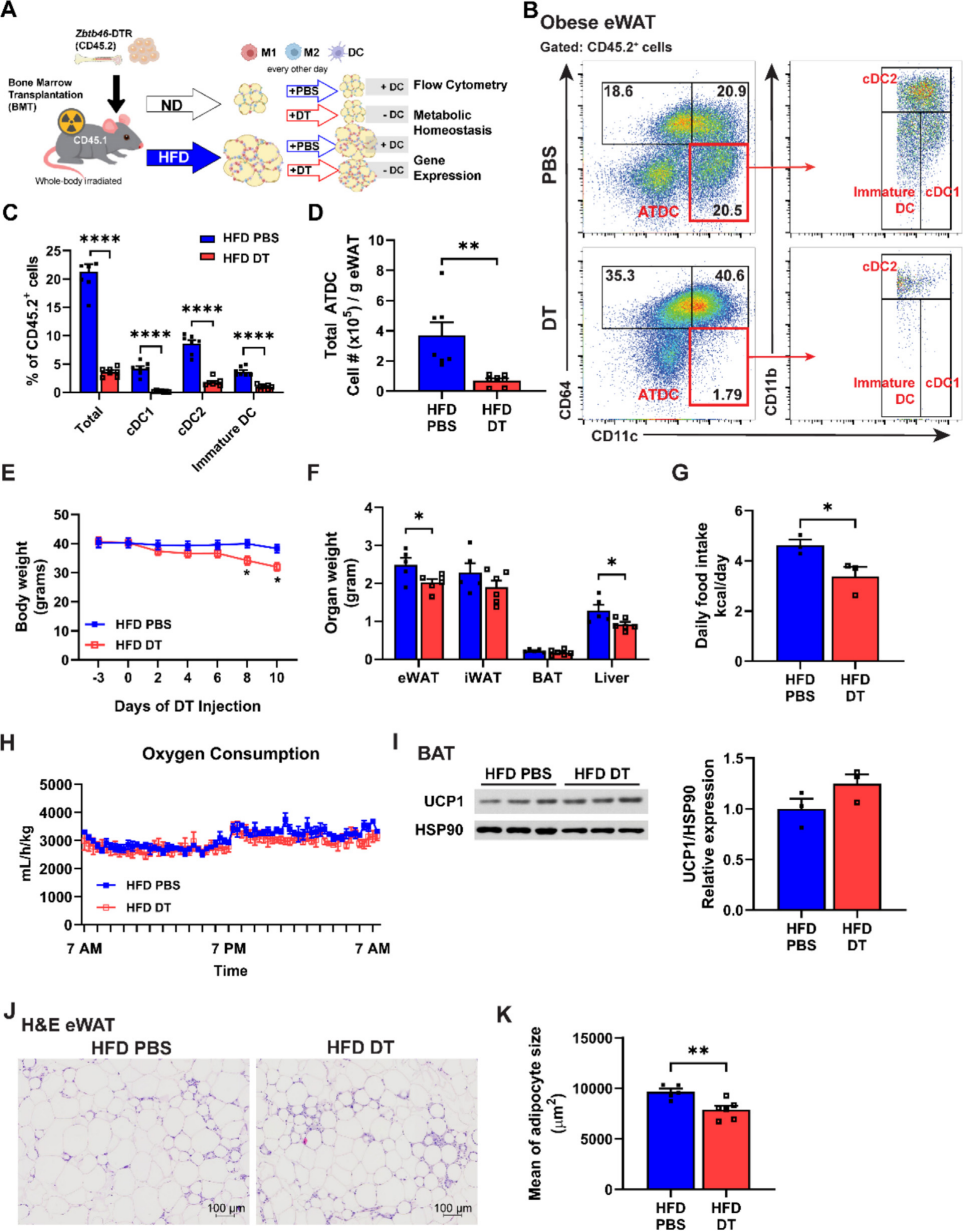
Inducible depletion of myeloid-derived Zbtb46^+^ cells improves metabolic parameters in obesity. (A) Schematic diagram for inducible depletion of adipose tissue dendritic cells (ATDCs) in obese chimeric *Zbtb46*-DTR mice using the diphtheria toxin (DT) injections. (B) Representatives flow cytometry diagram of donor-derived ATDCs (CD64^−^ CD11c^+^) including its CD11b subsets in epididymal white adipose tissue (eWAT) of obese chimeric mice after inducible depletion challenge. (C) Quantification of total ATDCs and its CD11b subsets, including cDC1 (CD11b^−^ CD11c^hi^), cDC2 (CD11b^+^), and immature DC (CD11b^−^ CD11c^+^) among leukocytes of donor cells (HFD PBS n = 6; HFD DT n = 7). (D) Quantitation of total ATDC cell number per gram of eWAT in PBS or DT-treated obese mice (HFD PBS n = 6; HFD DT n = 7). (E) Body weights during inducible depletion challenges with DT injections (HFD PBS n = 5; HFD DT n = 6). (F) Organ weights (HFD PBS n = 5; HFD DT n = 6). (G) Quantitation of daily food intake (n = 3 per group). (H) Oxygen consumption graphs at day 6 post DT challenge n = 3 per group). (I) Immunoblot of UCP1 protein in brown adipose tissue (BAT), and the relative expressions were quantified against HSP90 (n = 3 per group). (J) Representatives of H&E staining of eWAT. (K) Quantification of adipocyte size in eWAT (HFD PBS n = 5; HFD DT n = 6). Data are presented as mean ± SEM; *p < 0.05, ** *p* < 0.01, *** *p* < 0.001, ****p < 0.0001.

To investigate the role of myeloid-derived Zbtb46^+^ cells, chimeric *Zbtb46*-DTR mice were generated by transplanting *Zbtb46*-DTR bone marrow (CD45.2) into lethally irradiated wild-type recipients (CD45.1). Six weeks after reconstitution, chimeric mice were fed either a normal diet (ND) or high-fat diet (HFD) for 14 weeks, followed by intraperitoneal injections of PBS or diphtheria toxin (DT) every other day for two weeks (Fig. 1A). The depletion of donor-derived Zbtb46^+^ cells in obese epididymal white AT (eWAT) was confirmed by a reduction in the frequency of ATDCs from approximately 20% to less than 2% of leukocytes, including cDC1, cDC2, and immature DC subsets (Fig. 1B-C), as well as marked reduction in the total ATDC numbers (Fig. 1D). ATDC depletion was also observed in lean conditions (Fig. S1A–C). Additionally, depletion of other peripheral DCs, such as those in inguinal WAT (iWAT) and spleen, was detected (data not shown).

Following inducible depletion, ND-fed mice showed no changes in metabolic parameters, whereas HFD-fed obese mice exhibited significant reductions in body weight, eWAT mass, and liver weight (Fig. S1D–H; Fig. 1E-F). The reduction in body weight observed in obese Zbtb46^+^-depleted mice was not attributable to enhanced BAT thermogenesis but was mainly due to decreased food intake (Fig. 1G-I; Fig. S1F–H). In obese eWAT, a reduction in adipocyte size was also detected (Fig. 1J-K). Additionally, a decrease in fatty liver accumulation was noted, with significantly lower liver triglyceride levels (Fig. S2A–B). These findings suggest that inducible depletion of myeloid-derived Zbtb46^+^ cells effectively eliminates ATDCs, and two weeks of depletion reduces body weight by decreasing food intake, which is associated with improved obesity-related parameters.

**Fig. 2:**
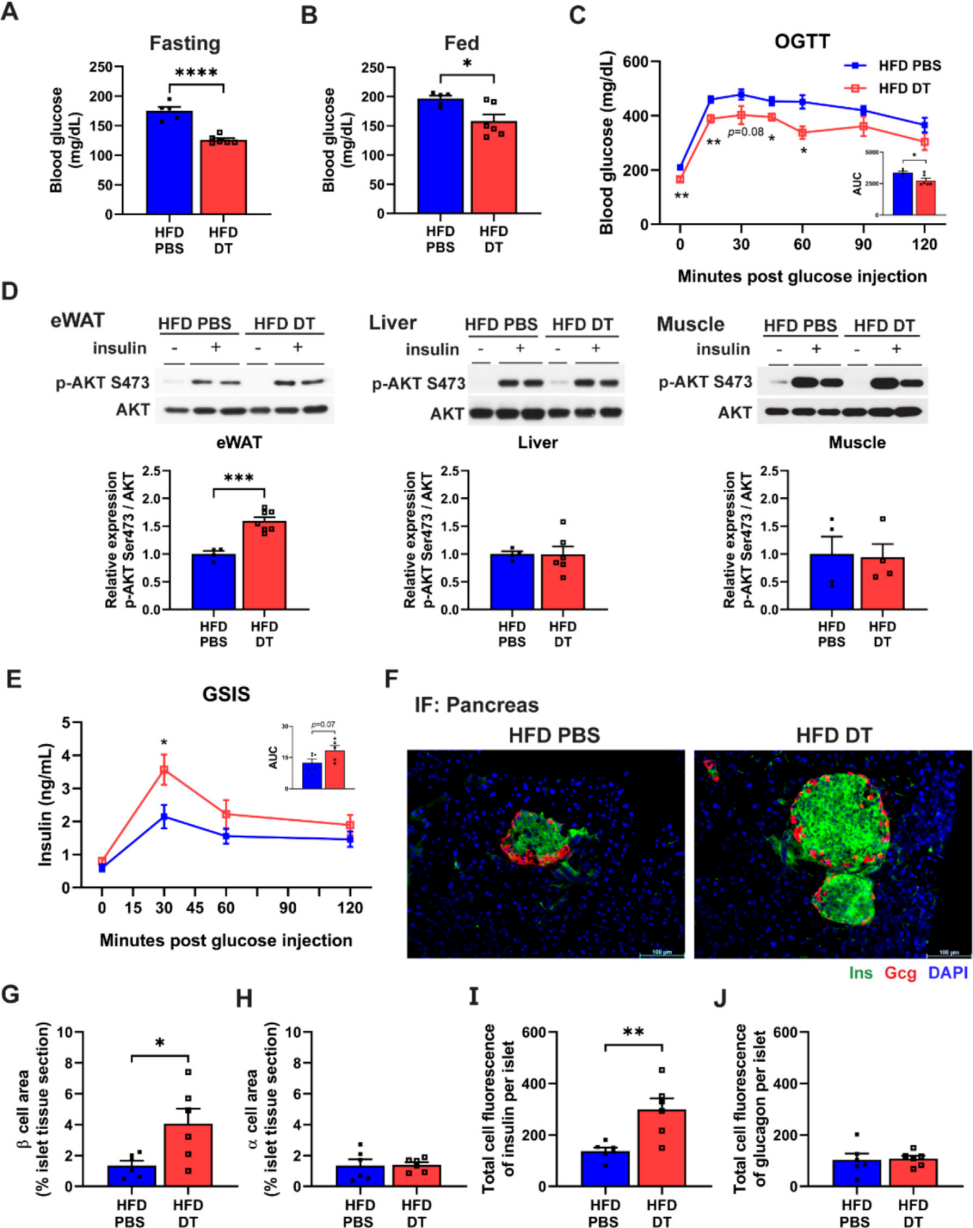
Inducible depletion of myeloid-derived Zbtb46^+^ cells improves glycemic control by enhancing adipose insulin sensitivity and increasing insulin secretion. (A–B) Fasting (16 h) blood glucose levels (A) and fed blood glucose levels (B) after the inducible depletion challenge (HFD PBS n = 5; HFD DT n = 6). (C) Blood glucose levels during oral glucose tolerance tests (OGTT) after a 16 h fasting and quantification of its area under curved (AUC) (HFD PBS n = 5; HFD DT n = 6). (D) Representative immunoblots of lysates from eWAT, liver, and muscle with anti-phosphorylated-AKT Ser473 or anti-AKT upon 15 min insulin challenge. The relative levels of p-AKT were normalized to AKT (eWAT: HFD PBS n = 4; HFD DT n = 7; Liver: HFD PBS n = 4; HFD DT n = 6; Muscle: HFD PBS n = 4; HFD DT n = 4). (E) Insulin levels during insulin during glucose-stimulated insulin secretion (GSIS) and quantification of its area under curved (AUC) (HFD PBS n = 6; HFD DT n = 5). (F) Representative immunofluorescence staining of Insulin^+^ pancreatic β-cells (green) and Glucagon^+^ pancreatic α-cells (red) from pancreas of PBS or DT-treated in obese *Zbtb46* chimeric mice. (G-H) Quantification of pancreatic β-cell area (G) and α-cell area (H) in percentage of total area of islet tissue section. One dot represents the average values from individual mouse (n = 6 per group). (I-J) Quantification of total cell fluorescence intensity of insulin (I) and glucagon (J) from each islet from six independent pancreas in each group (n = 6 per group). Data are presented as mean ± SEM; *p < 0.05, ** *p* < 0.01, *** *p* < 0.001, ****p < 0.0001.

To further assess the metabolic impact of myeloid-derived Zbtb46^+^ cell depletion, we next evaluated their glucose metabolism. Lean mice showed no change in fasting blood glucose levels after DT injection (Fig. S3), whereas obese mice exhibited significant improvements in glucose homeostasis, as evidenced by reduced fasting and fed blood glucose levels (Fig. 2A-B) and improved glucose tolerance (Fig. 2C). To investigate whether insulin sensitivity would be altered, we examined insulin signaling in metabolic active tissues including liver, muscle, and eWAT. Insulin-stimulated AKT phosphorylation was significantly elevated in eWAT, with no corresponding changes detected in the liver or muscle (Fig. 2D), indicating an AT-selective enhancement of insulin sensitivity. Glucose-stimulated insulin secretion (GSIS) assays further revealed a pronounced increase in postprandial insulin levels in DT-treated obese mice (Fig. 2E). Histological analysis showed an increase in pancreatic β-cell area and higher insulin fluorescence intensity without alterations in α-cell glucagon expression (Fig. 2F-J). Together, these results indicate that the inducible depletion of myeloid-derived Zbtb46^+^ cells in obesity enhances glucose homeostasis through improved AT insulin sensitivity and increased β-cell insulin secretion.

### Inducible depletion of myeloid-derived Zbtb46^+^ cells increases incretin levels and decreases DPP4 activity

**Fig. 3:**
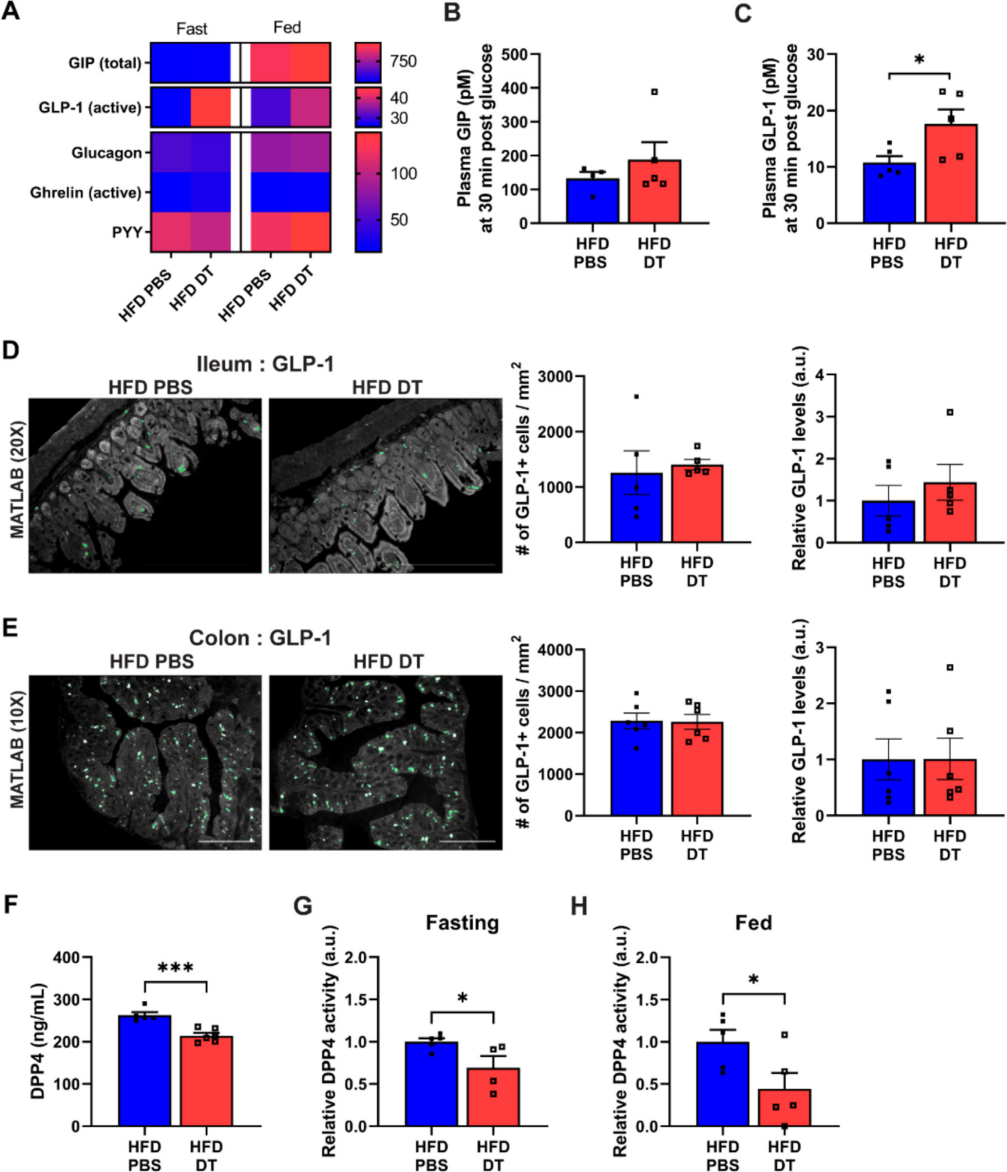
Inducible depletion of myeloid-derived Zbtb46^+^ cells elevates incretin levels and reduces DPP4 activity. (A) Expression of metabolic hormones in serum was measured by using MILLIPLEX platform after inducible depletion challenges in obese chimeric mice (n = 4 per group). (B) Postprandial plasma GIP levels (HFD PBS n = 4; HFD DT n = 5). (C) Postprandial plasma GLP-1 levels (n = 5 per group). (D-E) Representative MATLAB-based analysis of GLP-1^+^ immunofluorescence staining in the ileum (D) and colon (E) from PBS- or DT-treated obese *Zbtb46* chimeric mice. One dot represents the average values from individual mouse (ileum: n = 5 per group; colon: n = 6 per group). (F) DPP4 levels in the fasting plasma (HFD PBS n = 5; HFD DT n = 6). (G-H) Quantification of relative DPP4 activity in fasting (G) and fed conditions (H) (Fasting: HFD PBS n = 5; HFD DT n = 4; Fed: n = 5 per group). Data are presented as mean ± SEM; *p < 0.05, *** *p* < 0.001.

Following the observed increase in postprandial insulin levels, we examined the expression of metabolic hormones involved in regulating insulin secretion using the MILLIPLEX® Assay. Mild alterations in the levels of total GIP, glucagon, active ghrelin, and PYY expressions were observed under both fasting and fed conditions, while active GLP-1 levels were elevated in both states of Zbtb46^+^ cell deficiency (Fig. 3A). Notably, compared to controls, postprandial plasma incretin levels of DT-treated mice showed a 1.4-fold increase in total GIP and a marked 1.6-fold increase in total GLP-1 (Fig. 3B-C), indicating that elevated incretin levels could mediate the enhancement of insulin secretion from pancreatic β-cells [30].

To determine whether the increase in circulating GLP-1 levels reflected enhanced intestinal biosynthesis, we quantified GLP-1-positive (GLP-1^+^) L cells in the distal intestine using MATLAB-based image analysis (Fig. S4A). Immunostaining and quantification showed no change in L-cell density or per-cell GLP-1 immunoreactivity in either the ileum or colon (Fig. 3D-E). Co-staining for CD11c and GLP-1 revealed no colocalization and only occasional apposition, arguing against a consistent spatial association between DCs and L cells (Fig. S4B). Consistent with these findings, *ex vivo* primary ileal and colonic cultures from DT-treated mice released comparable amounts of GLP-1 to controls (Fig. S4C), indicating that DC depletion does not alter intestinal GLP-1 production. Pancreatic analysis likewise showed no changes in α-cell GLP-1 expression or signs of pancreatic inflammation (Fig. S4D–G), suggesting minimal impact of DC depletion on pancreatic sources of GLP-1. Since GLP-1 biosynthesis appeared unaltered, we measured circulating DPP4, the protease that degrades GLP-1. DT-treated obese mice exhibited significant reductions in serum DPP4 concentration and enzymatic activity under both fasting and fed conditions (Fig. 3F-H), consistent with elevated levels of incretins. Together, these data indicate that depletion of myeloid-derived Zbtb46^+^ cells increases circulating GLP-1 primarily via reduced systemic DPP4 activity rather than by enhanced intestinal GLP-1 production.

### Improvements in metabolic and glucose profiles following inducible depletion of myeloid-derived Zbtb46^+^ cells are not mediated by the inflammatory status of AT

The improvements in glucose homeostasis and insulin regulation prompted us to test whether inflammation would mediate these effects. Histological analysis showed that immune cell presence and ATMs forming crown-like structures (CLS) around adipocytes were markedly increased in eWAT from DT-treated mice (Fig. 1J; Fig. S5A). Flow cytometry analysis further revealed increased proportions of both pro-inflammatory (CD11c^+^) and anti-inflammatory (CD11c^−^) ATM subtypes (Fig. 1B; Fig. S5B–C). Gene expression analysis corroborated these findings, showing elevated mRNA levels of *Emr1* and *Mgl1* (Fig. S5D), alongside increased expression of pro-inflammatory cytokines (*Il6*, *Mcp1*, *Tnf*) and the anti-inflammatory cytokine *Il10* in eWAT (Fig. S5E). These results indicate that inducible depletion of ATDCs leads to a significant increase in ATMs, encompassing both pro- and anti-inflammatory ATM subtypes.

**Fig. 4:**
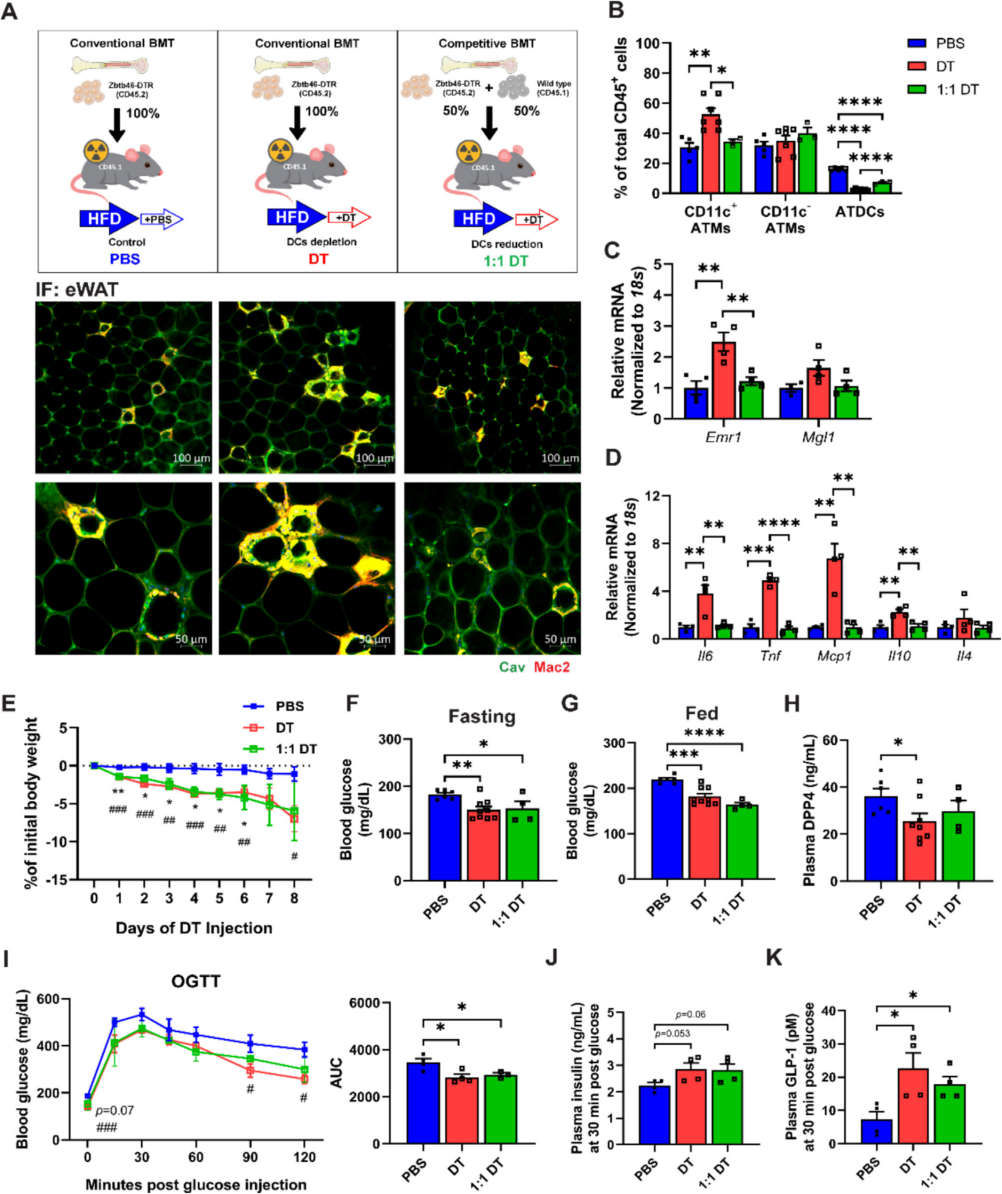
Improvements in glucose homeostasis following DC loss are independent from the adipose tissue inflammatory state. (A) Schematic illustration of conventional and competitive BMT experiment, and representative immunofluorescence staining of Mac2^+^ ATM in eWAT PBS, DT, and 1:1 DT group. (B) Frequencies of CD11c^+^ ATMs, CD11c^−^ ATMs, and ATDCs in percentages of total leukocytes (PBS n = 5; DT n = 7; 1:1 DT n = 3). (C–D) Relative mRNA expression of macrophages signature genes (C) and pro-inflammatory or anti-inflammatory cytokines (D) (n = 4 per group). (E) Body weight measurement during inducible depletion challenges in the percentages of initial body weight (* indicates significance between PBS and 1:1 DT; # indicates significance between PBS and DT; PBS n = 6; DT n = 9; 1:1 DT n = 4). (F) Fasting blood glucose levels and (G) fed blood glucose levels (PBS n = 6; DT n = 9; 1:1 DT n = 4). (H) DPP4 levels in the fasting plasma (PBS n = 6; DT n = 8; 1:1 DT n = 4). (I) Blood glucose levels during OGTT after a 16 h fasting and calculation of its AUC (PBS n = 4; DT n = 4; 1:1 DT n = 3). (J–K) Quantification of postprandial plasma insulin (J) and GLP-1 levels (K) (n = 4 per group). Data are presented as mean ± SEM; *p < 0.05, ** *p* < 0.01, *** *p* < 0.001, ****p < 0.0001.

It has been reported that deletion of specific immune cells induces other immune cells to compensate for loss [37]. To circumvent these challenges, we developed a competitive BMT model to partially deplete ATDCs (Fig. 4A). In this chimeric mouse model (1:1 DT), DT treatment reduced ATDCs by approximately 50 % and resulted in similar ATM numbers at CLS, with comparable CD11c^+^ ATM percentages to the PBS group (Fig. 4A-B). This normalization of ATM accumulation was confirmed by mRNA expression levels of *Emr1* and *Mgl1*, as well as pro- and anti-inflammatory cytokine mRNA levels, all of which were comparable to controls (Fig. 4C-D). Intriguingly, both partial reduction and total depletion of myeloid-derived Zbtb46^+^ cells consistently improved obesity-associated glucose homeostasis (Fig. 4E-G), indicating that metabolic regulation occurs independently of ATM profiles or AT inflammation. Notably, the 1:1 DT group also showed significant improvements in DPP4-incretin-insulin regulation, as evidenced by reduced plasma DPP4 levels, enhanced glucose tolerance, improved insulin secretion, and elevated postprandial GLP-1 levels (Fig. 4H-K). Taken together, these findings highlight the predominantly inflammation-independent role of myeloid-derived Zbtb46^+^ cells in regulating metabolic parameters in obesity, likely through modulation of DPP4-incretin effects.

### Acute weight loss in Zbtb46^+^ cell-ablated mice does not affect systemic glucose homeostasis while enhancing tissue insulin sensitivity

**Fig. 5:**
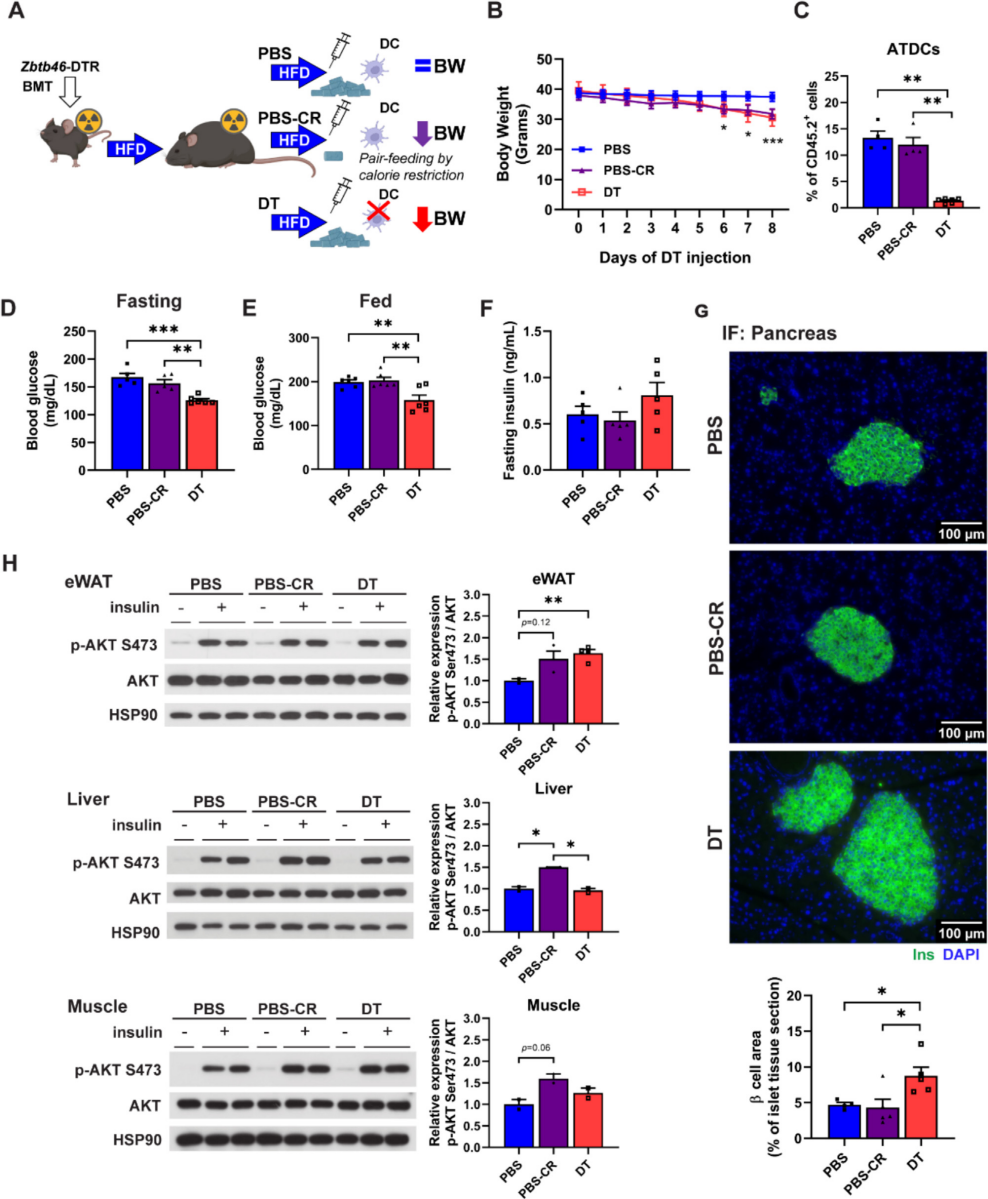
Acute weight loss in Zbtb46^+^ cell-depleted mice does not impact systemic glucose homeostasis but improves tissue insulin sensitivity. (A) Schematic illustration of experimental design of BMT-pair feeding model by calorie restriction (CR) during DT challenge. (B) Body weight measurement during CR and/or DT challenge (n = 6 per group). (C) Quantification of ATDCs percentage in eWAT among leukocytes of donor cells after CR and/or DT challenge. (D) Fasting blood glucose levels (PBS n = 5; PBS-CR n = 5; DT n = 6). (E) Fed blood glucose levels (n = 6 per group). (F) Fasting insulin levels were measured after CR and/or DT challenge (n = 5 per group). (G) Representative immunofluorescence staining of Insulin^+^ from the pancreas and the quantification of β-cell area in the pair feeding-DT challenge model. One dot represents the average values from individual mouse (PBS n = 4; PBS-CR n = 5; DT n = 5). (H) Representative immunoblots of lysates from eWAT, liver, and muscle against anti-p-AKTSer473 and anti-AKT, and their relative quantitation (eWAT: PBS n = 2; PBS-CR n = 3; DT n = 4; Liver and Muscle: n = 2 per group). Data are presented as mean ± SEM; *p < 0.05, ** *p* < 0.01, *** *p* < 0.001.

To ascertain whether the alteration of glucose homeostasis was a primary defect or secondary to obesity, we conducted subsequent studies using a pair-feeding experiment. In addition to PBS or DT injections, a calorie restriction (CR) protocol was applied to the PBS group, creating a weight-matched PBS-CR group to the DT group (Fig. 5A-B). Weight matching in the PBS-CR group did not affect the ATDC population in eWAT (Fig. 5C). While the DT group consistently showed improvements in glucose homeostasis, the PBS-CR group exhibited no significant changes in fasting or fed blood glucose levels, similar to the PBS group. This indicates that the improved systemic glucose homeostasis in the DT group was independent of acute weight loss (Fig. 5D-F). Moreover, the pancreatic β-cell area in the PBS-CR group was comparable to that in the control group (Fig. 5G), suggesting that the enhanced insulin production in the DT group was not driven by acute weight loss. Insulin signaling analysis further revealed that acute weight loss improved insulin sensitivity in eWAT, liver, and muscle (Fig. 5H). Notably, the increase in insulin-stimulated AKT phosphorylation in eWAT in the PBS-CR group was comparable to that in the DT group, suggesting that acute weight loss may partially contribute to the enhanced AT insulin sensitivity observed with Zbtb46^+^ cell depletion. Altogether, these results demonstrate that glucose control in the absence of myeloid-derived Zbtb46^+^ cells is irrespective of body weight.

### Adipose tissue dendritic cells (ATDCs) contribute to reduced DPP4 activity in Zbtb46^+^ cell-depleted mice

**Fig. 6:**
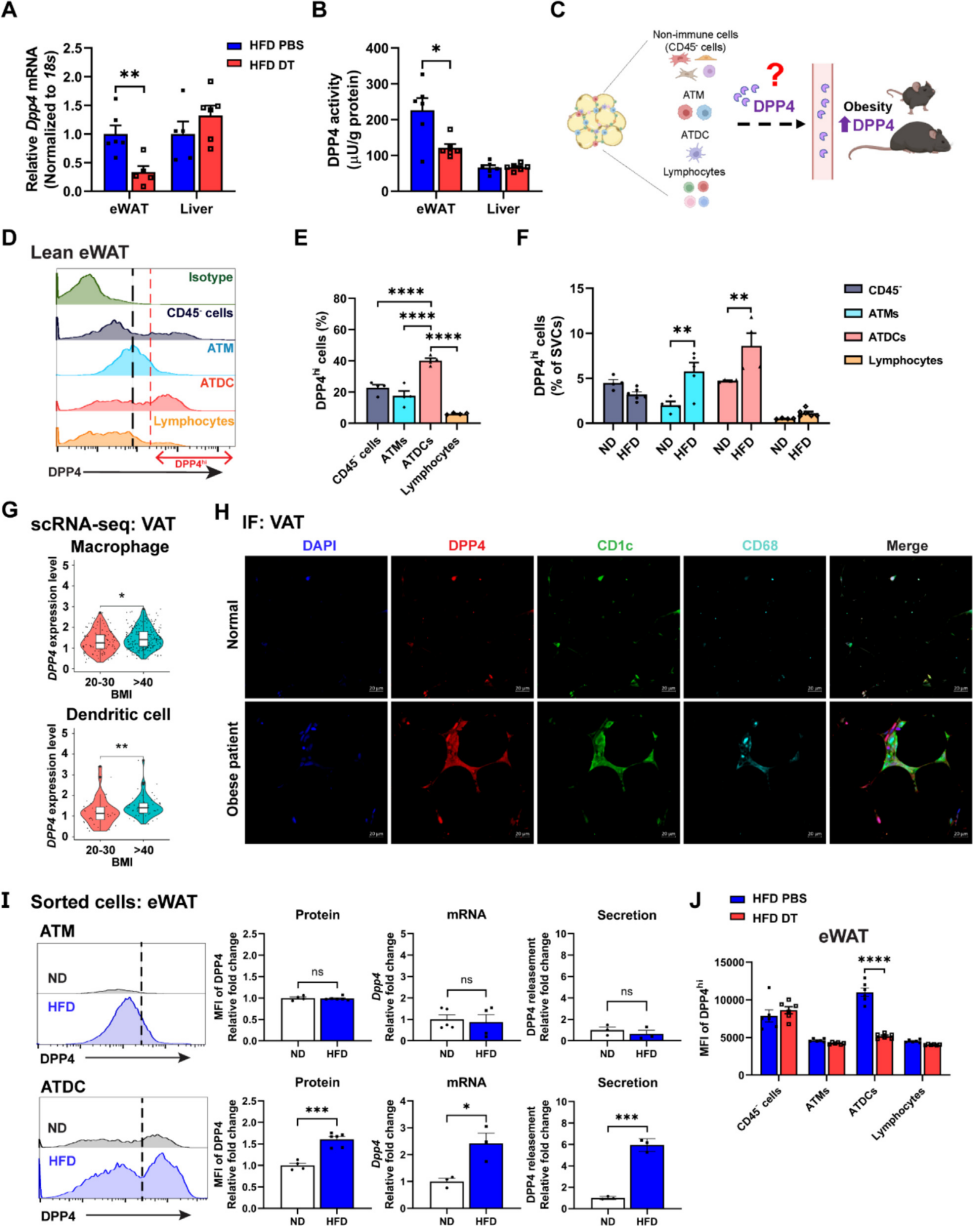
Adipose tissue dendritic cells in visceral fat are key contributors to DPP4 expression and secretion in obesity. (A) Relative mRNA expression of *Dpp4* in eWAT and liver tissue (n = 5–6 per group). (B) DPP4 activity levels in eWAT and liver tissue (n = 6 per group). (C) Schematic illustration of DPP4 expression in ATDCs and its potential contribution to systemic regulation, among other cells within stromal vascular cells (SVCs). (D–F) C57BL/6J mice were induced to obesity by 16 weeks of HFD and expressions of DPP4 in eWAT immune cells were evaluated (ND n = 4; HFD n = 6). (D) Representative histogram and (E) quantification of DPP4^hi^ expression in CD45^−^ cells, ATMs (CD64^+^), ATDCs (CD64^−^ CD11c^+^), and lymphocytes (CD3^+^) from lean visceral eWAT. (F) Quantification of DPP4^hi^-expressing cells in percentages of total SVCs. (G) *DPP4* expression in macrophages and dendritic cells from human visceral AT (VAT) by single-cell RNA sequencing (scRNA-seq) analysis. (H) Representative immunofluorescence (IF) staining of DPP4 (red), CD1c (green), CD68 (turquoise), and DAPI (blue) in visceral AT (VAT) sections from normal (n = 3) and obese patients (n = 3). (I) Representative histogram and relative quantification of DPP4 median fluorescence intensity (MFI) (ND n = 4; HFD n = 6), mRNA, and secretion levels from sorted ATMs and ATDCs in lean and obese conditions (n = 3–5 per group). (J) Quantification of DPP4^hi^ MFI in CD45^−^ cells, ATMs (CD64^+^), ATDCs (CD64^−^ CD11c^+^), and lymphocytes (CD3^+^) from obese chimeric *Zbtb46*-DTR mice following the inducible depletion of DCs (n = 6 per group). Data are presented as mean ± SEM; *p < 0.05, ** *p* < 0.01, *** *p* < 0.001, ****p < 0.0001.

Based on the findings of enhanced DPP4-incretin-insulin regulation following the inducible loss of myeloid-derived Zbtb46^+^ cells, we sought to investigate the mechanism underlying the reduction in DPP4 activity. First, we examined tissue source of circulating DPP4 in obesity under conditions of myeloid Zbtb46^+^ cell deficiency. Consistent with previous reports identifying DPP4 as both an adipokine and a hepatokine in obesity [[38], [39], [40]], plasma DPP4 activity and DPP4 expression were elevated in obese eWAT and liver, but remained unchanged in iWAT (Fig. S6A–D). Following the inducible depletion of DCs in obesity, DPP4 mRNA expression was significantly downregulated only in eWAT, with no changes in liver, iWAT, pancreas, or skeletal muscle (Fig. 6A; Fig. S6E–G). Marked reductions in eWAT DPP4 mRNA expression were also observed in the 1:1 DT group, suggesting that both partial and total DC depletion decrease AT-DPP4 expression (Fig. S6H). Consistently, DPP4 enzymatic activity was significantly reduced exclusively in eWAT, but not in the liver (Fig. 6B), indicating that eWAT is the primary tissue modulating DPP4 activity upon loss of myeloid-derived Zbtb46^+^ cells. Additionally, the DPP4 protein levels in the colon were unaltered (Fig. S6I), substantiating that GLP-1 regulation is affected post-secretion via altered degradation rather than increased intestinal biosynthesis.

Second, to define the cellular source(s) of DPP4 within eWAT, we measured DPP4 expression by flow cytometry in adipose stromal vascular fraction subsets—including ATMs, ATDCs, lymphocytes, and non-immune cells— from both lean and obese mice (Fig. 6C). Among these, ATDCs exhibited the highest percentage of DPP4^hi^ expression, followed by CD45^−^ cells, ATMs, and lymphocytes (Fig. 6D-E). In total SVCs, lean ATDCs and CD45^−^ cells showed similar frequencies of DPP4^hi^ cells. However, in obesity, the frequency of DPP4^hi^ cells notably increased in ATDCs and, to a lesser extent, in ATMs, with no significant changes observed in CD45^−^ cells and lymphocytes (Fig. 6F). These data identify ATDCs as the predominant immune cell population expressing DPP4 in eWAT under both steady-state and obese conditions and suggest that ATDC loss is a major driver of reduced adipose DPP4.

To assess translational relevance, we analyzed human visceral AT (VAT). Public single-cell RNA-seq datasets showed higher *DPP4* expression in DCs (*ITGAX^+^*) and macrophages (*ITGAM^+^*, *CD14^+^*) from individuals with obesity, with the increase being most robust in DCs (Fig. S6J; Fig. 6G). Immunofluorescence of obese VAT confirmed elevated DPP4 staining, particularly within CLS regions where DPP4 co-localized predominantly with both CD1c^+^ ATDCs and CD68^+^ ATMs (Fig. 6H). Thus, ATDCs are the major cellular source of DPP4 in obese human VAT, consistent with our murine observations.

Finally, we directly tested DPP4 expression and secretion in sorted ATDCs from lean and obese eWAT, using ATMs as a comparison. Notably, only obese ATDCs exhibited significantly elevated DPP4 levels, as measured by median fluorescence intensity (MFI) and mRNA expression. Obese ATDC cultures also showed increased DPP4 protein release into the media, whereas no such change was observed in obese ATMs (Fig. 6I). The role of ATDCs as the primary sources of DPP4 in obese eWAT was further substantiated by a specific reduction in DPP4 expression upon ATDC depletion, with no changes observed in other cell types (Fig. 6J). DC populations in other tissues (iWAT and spleen) did not represent major DPP4-expressing populations (data not shown), and their depletion did not alter DPP4 expression in immune cells (Fig. S6K). Collectively, these results demonstrate that inducible loss of ATDCs in eWAT is the primary mechanism underlying reduced adipose and systemic DPP4 in obese chimeric *Zbtb46*-DTR mice.

### Enhanced GLP-1/GLP-1 receptor signaling improves glucose tolerance in Zbtb46^+^ cell-depleted mice

**Fig. 7:**
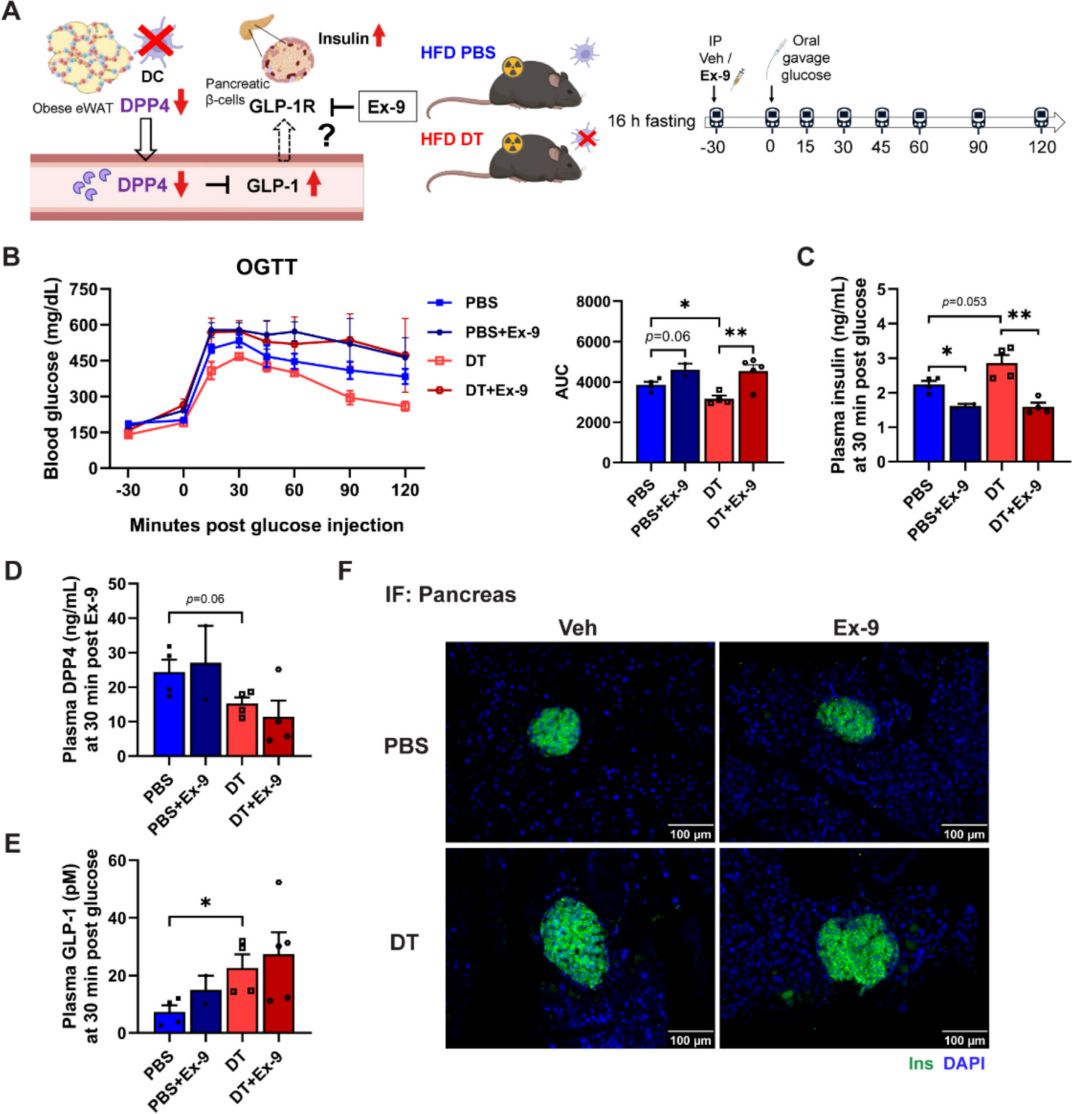
Enhanced GLP-1 action through the GLP-1 receptor (GLP-1R) mediates improved glycemic control. (A) Schematic illustration of Exendin-9 (Ex-9; 2ug/g BW) action and treatment intraperitoneally injected 30 min prior to OGTT challenges. (B) Blood glucose levels during OGTT and its AUC was calculated (PBS n = 4; PBS + Ex-9 n = 2; DT n = 4; DT + Ex-9 n = 5). (C–E) Postprandial plasma insulin levels (C), DPP4 levels (D), GLP-1 levels (E) in vehicles or Ex-9 treatment in PBS and DT groups (PBS n = 4; PBS + Ex-9 n = 2; DT n = 4; DT + Ex-9 n = 4–5). (F) Representative immunofluorescence staining of Insulin^+^ pancreatic β-cell in pancreas tissue of PBS or DT group three days post treatment of Ex-9. Data are presented as mean ± SEM; *p < 0.05, ** *p* < 0.01.

To examine whether increased incretin levels, particularly GLP-1, directly mediate enhanced glucose tolerance through incretin effects, we treated mice with a single dose of GLP-1 receptor antagonist Exendin-9 (Ex-9) prior to glucose administration (Fig. 7A). Remarkably, Ex-9 treatment completely abolished the improved glucose tolerance observed in the DT group after inducible depletion, indicating that GLP-1/GLP-1R is required for regulating glucose tolerance following Zbtb46^+^ cell depletion (Fig. 7B). Moreover, inhibiting GLP-1/GLP-1R pathway markedly reduced postprandial insulin levels until reaching similar levels in both the PBS and DT groups, suggesting that incretin effects mediated by GLP-1 regulate insulin secretion and glucose homeostasis (Fig. 7C). There were no alterations observed in plasma DPP4 or postprandial GLP-1 levels after Ex-9 treatment in either group (Fig. 7D-E). Additionally, single dose treatment of Ex-9 did not affect pancreatic insulin expression or eWAT profiles (Fig. 7F; Fig. S7A–C). Overall, these findings indicate that enhanced GLP-1 action via the GLP-1R directly mediates improved glucose tolerance in obese chimeric *Zbtb46*-DTR mice, acting as the primary mechanism regulating incretin effects on insulin secretion after the inducible depletion of DPP4-expressing ATDCs in obesity.

### DPP4 in DCs regulates GLP-1–induced insulin secretion

**Fig. 8:**
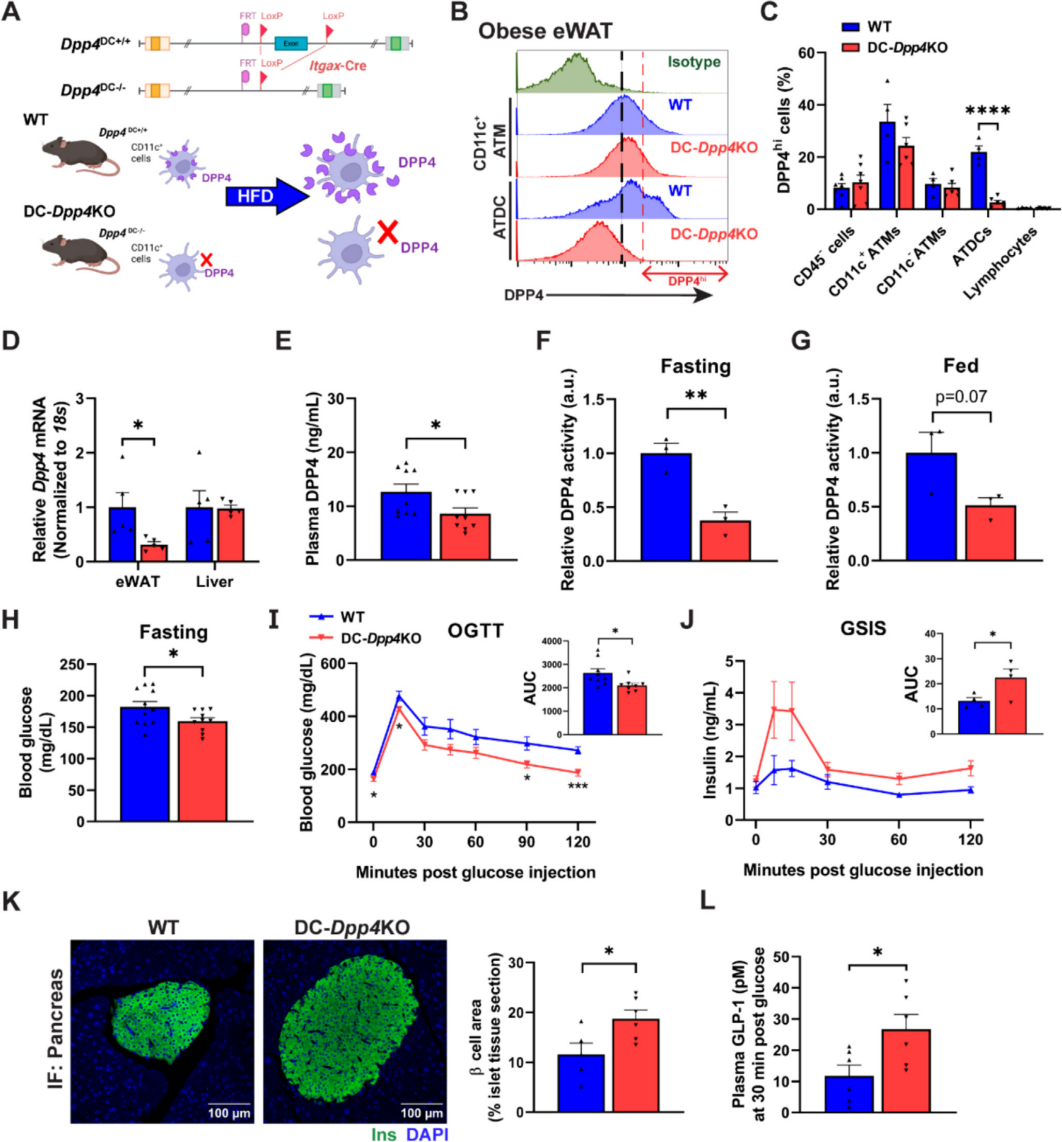
Deficiency of DPP4 in dendritic cells reduces systemic DPP4 activity and enhances incretin effects. (A) Schematic illustration of HFD challenge in DC-*Dpp4*KO (*Dpp4*^DC−/−^) mice and their wild-type (WT;*Dpp4*^DC+/+^) littermates. (B) Representative histogram of DPP4^hi^ expression in CD11^+^ ATMs and ATDCs from obese eWAT. (C) Quantification of DPP4^hi^ frequency in CD45^−^ cells, CD64^+^ CD11c^+^ ATMs, CD64^+^ CD11c^−^ ATMs, ATDCs (CD64^−^ CD11c^+^), and lymphocytes (CD3^+^) from obese eWAT (WT n = 4; DC-*Dpp4*KO n = 6). (D) Relative *Dpp4* mRNA expression in eWAT and liver (n = 5 per group). (E) Plasma DPP4 levels under fasting conditions (n = 9 per group). (F–G) Plasma DPP4 activity under fasting (F) and fed (G) conditions (n = 3 per group). (H) Fasting blood glucose levels (WT n = 11; DC-*Dpp4*KO n = 10). (I) Blood glucose levels during oral glucose tolerance tests (OGTT) after a 16 h fasting and quantification of its area under curved (AUC) (WT n = 9; DC-*Dpp4*KO n = 8). (J) Plasma insulin levels during glucose-stimulated insulin secretion (GSIS) and corresponding AUC (n = 4 per group). (K) Representative immunofluorescence staining of Insulin^+^ pancreatic β-cells (green) and area quantification in WT and DC-*Dpp4*KO obese mice. One dot represents the average values from individual mouse (WT n = 5; DC-*Dpp4*KO n = 6). (L) Postprandial plasma GLP-1 levels (n = 6 per group). Data are presented as mean ± SEM; *p < 0.05, ** *p* < 0.01, ****p < 0.0001.

To confirm whether DPP4 expression in DCs directly mediates enhanced GLP-1 signaling, we generated DC-specific DPP4 knockout mice (DC-*Dpp4*KO) and subjected them to HFD feeding (Fig. S8A; Fig. 8A). DC-*Dpp4*KO mice gained body weight and had organ weights comparable to control littermates (Fig. S8B–C). Flow cytometry analysis confirmed reduced DPP4 expression in both CD11c^+^ adipose immune populations, with a significant reduction in ATDCs and only a minor change in CD11c^+^ ATMs (Fig. 8B-C), consistent with an ATDC-restricted depletion. Concordantly, DPP4 mRNA was markedly reduced in eWAT but unchanged in liver, and both circulating DPP4 concentrations and enzymatic activity were significantly lower in DC-*Dpp4KO* mice (Fig. 8D-G). Notably, DC-*Dpp4*KO mice recapitulated the metabolic phenotype observed after Zbtb46^+^ cell depletion: they displayed improved glucose tolerance, increased insulin secretion, enlarged pancreatic β-cell area, and elevated postprandial plasma GLP-1 levels (Fig. 8H-L). Collectively, these data strongly support ATDC-derived DPP4 as a major contributor to circulating DPP4 and a functional regulator of the GLP-1/insulin axis in diet-induced obesity.

## Discussion

This study is the first to demonstrate that non-inflammatory functions of DCs play a crucial role in regulating glucose homeostasis and energy balance in established obesity, as shown by the inducible depletion of myeloid-derived Zbtb46^+^ cells. Notably, improvements in glycemic control were observed even in weight-matched mice and in the presence of increased ATM accumulation, indicating that these effects were not attributable to weight loss or the inflammatory state of AT. Moreover, enhanced glucose homeostasis was primarily attributed to increased GLP-1 levels, which upregulated insulin secretion and reduced food intake. Mechanistically, GLP-1 induction was linked to decreased DPP4 activity, resulting from the depletion of ATDCs, a key source of circulating DPP4. DC-specific DPP4 deficiency further demonstrated that DPP4 expression in ATDCs substantially regulates GLP-1 availability and its incretin effects on insulin secretion in obesity. These findings strongly indicate that the DPP4/GLP-1/GLP-1R axis is regulated by ATDCs, and that their non-inflammatory function plays a pivotal role in regulating obesity-associated glucose homeostasis.

Our findings reveal a previously underappreciated non-inflammatory role of ATDCs in regulating glycemic control in established obesity. While most research has focused on ATDCs as key drivers of AT inflammation during obesity progression [8,41], their precise regulatory mechanisms throughout the entire course of obesity development remain insufficiently explored. Animal studies using whole-body DC depletion models, such as *Flt3l*^−^*^/^*^−^, *Csf2*^−^*^/^*^−^*,* and *Ccr7*^−^*^/^*^−^, have shown that ATDC deficiency reduces AT inflammation, limits weight gain, and improves glucose and insulin resistance, supporting the role of ATDC-mediated inflammatory functions in metabolic dysregulation during obesity progression [13,[17], [18], [19], [20]]. However, these findings do not establish ATDC as a viable therapeutic target for existing obesity. The differential dynamics of ATDC during obesity—where ATDC numbers initially rise during the first eight weeks of HFD feeding and then are sustained in established obesity [8]—suggest that ATDCs may exhibit distinct functional properties depending on the stage and chronicity of obesity [8,41]. Here, we demonstrate that short-term (2 weeks) depletion of ATDCs in established obesity reduces body weight and improves glucose homeostasis via a predominantly non-inflammatory mechanism, independent of the inflammatory state of AT. The beneficial effects of ATDC depletion in established obesity underscore the critical role of ATDCs across all obesity stages and their potential as a novel therapeutic target for obesity intervention. Notably, body weight reduction following ATDC depletion was also observed in various DC-deficient experimental models, suggesting that ATDCs’ non-inflammatory functions play a significant role in regulating obesity progression and associated metabolic disturbances. However, despite this link to weight regulation, the glucose-regulatory function of ATDCs in this model appears to be independent of body weight reduction, further emphasizing their distinct metabolic role in established obesity. Moreover, while our data emphasize the glucoregulatory role of ATDCs in established obesity via a non-inflammatory pathway, we cannot fully exclude the possibility that ATDCs may also exert inflammatory effects on glucose homeostasis, particularly under chronic depletion conditions.

Given the current strategy of targeting the incretin effect for obesity treatment, DPP4 has been identified as a key molecule regulating the GLP-1/GLP-1R pathway, the central mechanism of the incretin effect. However, the specific cells within metabolic tissues that predominantly contribute to the *in vivo* function of GLP-1 remain unclear, as multiple cell types and tissues have been implicated in regulating GLP-1 activity [40,42]. While hepatic DPP4 has been reported to influence plasma DPP4 levels in obesity, we observed no changes in hepatic DPP4 expression or activity, despite improved glucose homeostasis and elevated circulating GLP-1 levels. These findings align with previous studies demonstrating the minimal role of hepatic DPP4 in glucose homeostasis and inconsistent effects on incretin levels [40,43,44]. Instead, our study identifies adipose DPP4 activity as significantly correlated with circulating GLP-1 levels and glucose regulation, emphasizing the role of visceral fat in modulating DPP4 and incretin pathways. Among the various cell types in VAT, our findings clarify that ATDCs, rather than ATMs, serve as the primary source of DPP4 in AT as determined using specific ATM and ATDC markers [32]. Importantly, ATDC-derived DPP4 controls the systemic DPP4/GLP-1/GLP-1R axis, reinforcing a prominent role for eWAT-DPP4 in obesity, whereas adipocyte-derived DPP4 has only a minimal effect on systemic incretin action [40,42]. The identification of ATDC as the major DPP4-expressing population is further supported by human VAT single-cell and immunofluorescence data. Nonetheless, our model broadly targets cDCs and cannot discriminate between cDC1 and cDC2, nor fully exclude contributions from pDCs and moDCs [34,35,45]. The specificity of ATDC-derived DPP4 in obesity, particularly in eWAT compared to other tissue DC-DPP4, suggests that obesity-associated conditions may uniquely shape ATDC subsets and their metabolic function. Future studies employing higher-resolution approaches will be necessary to delineate the DPP4^hi^-ATDC subsets, identify cues regulating ATDC-DPP4 expression, and establish their translational relevance to human DC heterogeneity.

The GLP-1/GLP-1R axis is a well-established regulator of glucose metabolism and appetite control [46,47]. In pancreatic β-cells, GLP-1 signaling not only promotes insulin secretion but also enhances β-cell function and proliferation [47]. Consistent with this, our study demonstrates that GLP-1 activation by ATDC depletion improves islet insulin secretion in obesity, independently of body weight reduction. This highlights the early-stage regulatory effects of incretin-based therapies and underscores the potent role of the GLP-1 pathway in glycemic control. Beyond pancreatic function, GLP-1R signaling in the central nervous system (CNS) plays a crucial role in energy balance and glucose metabolism [48]. Although primarily recognized for its appetite-suppressing effects, CNS GLP-1R activation has also been implicated in direct metabolic regulation [49]. Furthermore, GLP-1R signaling in peripheral tissues, such as liver and skeletal muscle, has been suggested to influence systemic glucose homeostasis [47]. These observations indicate that both indirect CNS–peripheral crosstalk and direct GLP-1R–mediated actions in obesity-associated metabolic tissues warrant further investigation to elucidate their integrated roles in glucose regulation. Notably, emerging evidence suggests that DCs express GLP-1R [50], and both clinical and animal studies have reported weight-independent effects of GLP-1R agonists, including inflammation suppression [51]. These findings raise the intriguing possibility that DCs may serve as mediators of GLP-1 signaling in adipose tissue, linking the incretin pathway to immune-metabolic regulation in obesity.

In the present study, augmented GLP-1–stimulated insulin secretion emerged as the dominant mechanism improving whole-body glucose tolerance, but we cannot exclude a contributory role for selective improvements in AT insulin sensitivity (Fig. 2D). Several non-mutually exclusive mechanisms could explain how ATDC/DPP4 modulation differentially affects AT insulin action. First, loss of ATDC-derived DPP4 raised circulating active incretins, and although adipocyte GLP-1R expression is low, both direct GLP-1 effects on glucose uptake/lipolysis and indirect effects such as enhanced AT capillarization may locally improve adipocyte insulin responsiveness [[52], [53], [54]]. Second, high DPP4 in ATDCs suggests paracrine modulation of adipocyte signaling, potentially via caveolin-1 interactions [55], such that reductions in local DPP4 alter adipocyte-insulin signaling independently of systemic GLP-1. Third, GLP-1–independent mechanisms—such as altered adiposity, changes in other gut hormones (GIP, PYY), or additional ATDC-derived factors—may also contribute. The comparable improvements in AT insulin sensitivity with weight loss argue for an indirect component, whereas the recapitulation of metabolic benefits in DC-specific *Dpp4* knockout mice supports a substantive, ATDC-centered mechanism. Overall, the data favor a model in which enhanced insulin secretion (via reduced systemic DPP4 and increased active GLP-1) is the primary driver of improved glycemia, with concurrent local AT effects arising from a combination of incretin action, reduced local DPP4, and other ATDC-dependent factors. Further work is needed to dissect these components.

## Conclusion

In summary, this study uncovers a predominant non-inflammatory role of ATDCs in regulating glycemic control in obesity via the DPP4/GLP-1/GLP-1R axis. Our findings deepen the understanding of ATDCs’ functions within the complex microenvironment of obese AT, emphasizing their metabolic regulatory roles beyond traditional inflammatory pathways. Moreover, we introduce ATDCs as critical modulators of DPP4/GLP-1 pathway in obesity, offering valuable insights for developing therapeutic strategies targeting obesity-related metabolic disorders. This work underscores the broader implications of immune cell-mediated regulation of metabolism, opening new avenues for targeted interventions in metabolic diseases.

## Compliance with ethics requirements

All Institutional and National Guidelines for the care and use of animals (fisheries) were followed.

## Declaration of competing interest

The authors declare that they have no known competing financial interests or personal relationships that could have appeared to influence the work reported in this paper.
