# Supplementary material for: Depletion of myeloid-derived Zbtb46+ cells improves glycemic control in obesity via the DPP4/GLP-1 pathway
Source: J Adv Res. 2025 Oct 23;85:883–98. doi: 10.1016/j.jare.2025.10.032 (PMC13316555; doi:10.1016/j.jare.2025.10.032)
Supplement: Supplementary Data 1 [file mmc1.docx]

**
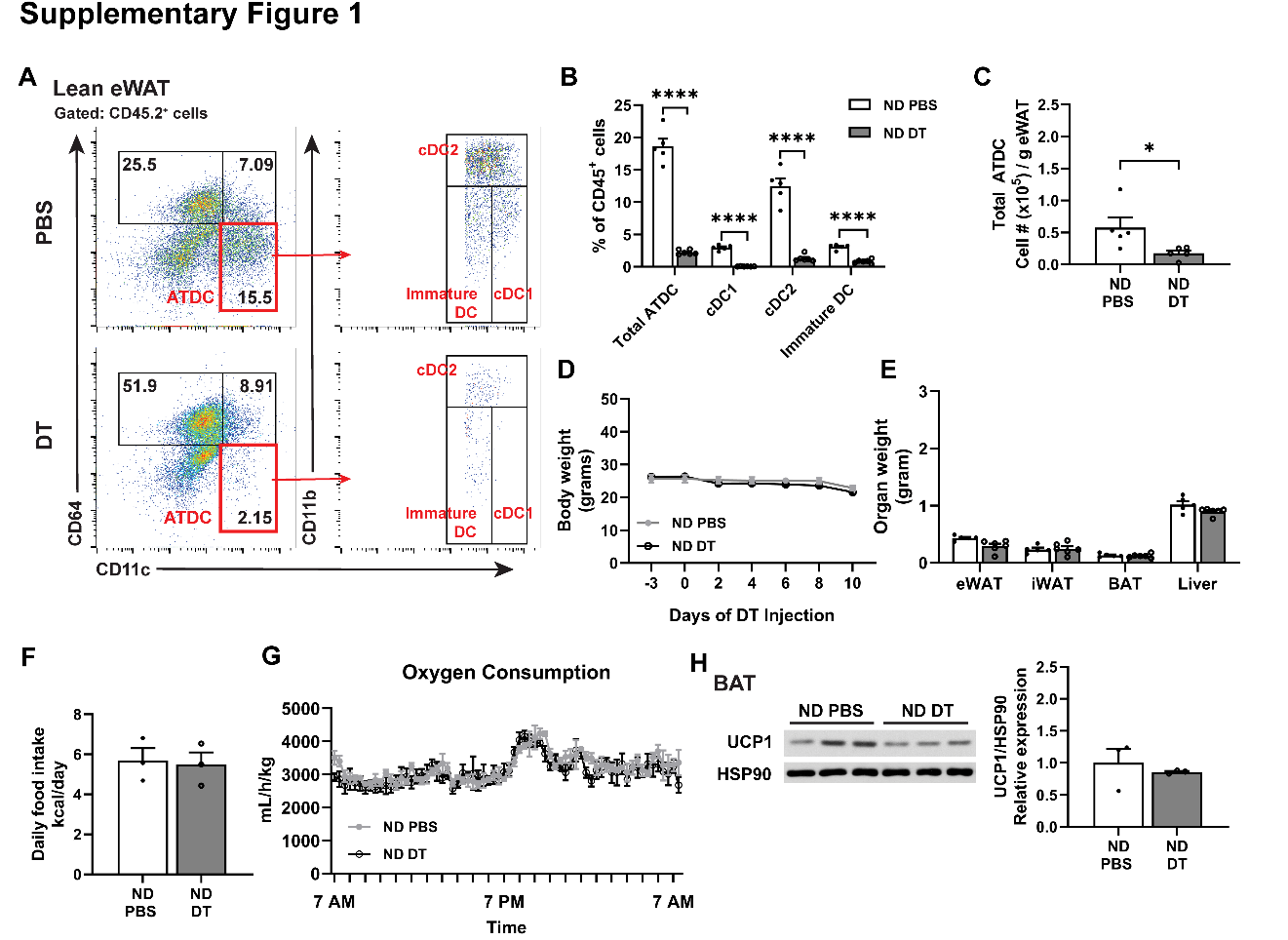
**

**Figure S1. Inducible depletion of myeloid-derived Zbtb46^+^ cells does not affect metabolic parameters in lean mice.** (A) Representatives flow cytometry diagram of donor-derived ATDCs (CD64^−^ CD11c^+^) including its CD11b subsets in epididymal white adipose tissue (eWAT) of lean chimeric mice after inducible depletion challenge. (B) Quantification of total ATDCs and its CD11b subsets, including cDC1 (CD11b^−^ CD11c^hi^), cDC2 (CD11b^+^), and immature DC (CD11b^−^ CD11c^+^) among leukocytes of donor cells (ND PBS n=5; ND DT n=6). (C) Quantitation of total ATDC cell number per gram of eWAT in PBS or DT-treated obese mice (ND PBS n=5; ND DT n=6). (D) Body weights during inducible depletion challenges with DT injections (ND PBS n=5; ND DT n=6). (E) Organ weights (ND PBS n=5; ND DT n=6). (F) Quantitation of daily food intake (n=3 per group). (G) Oxygen consumption graphs at day 6 post DT challenge (n=3 per group). (H) Immunoblot of UCP1 protein in brown adipose tissue (BAT), and the relative expressions were quantified against HSP90 (n=3 per group). Data are presented as mean ± SEM; **p*< 0.05, *****p*<0.0001.


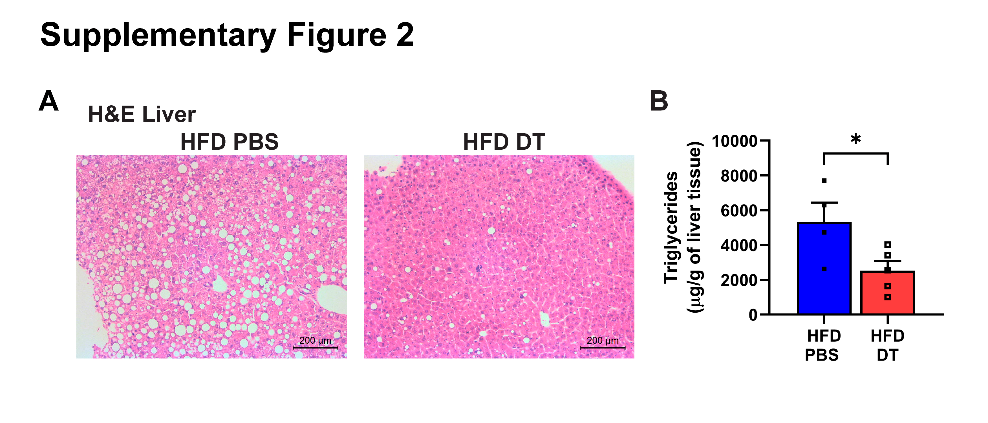


**Figure S2. Inducible depletion of myeloid-derived Zbtb46^+^ cells reduces hepatic fat accumulation in obese mice.** (A) Representatives of H&E staining of liver. (B) Quantification of triglycerides amount in liver tissue (HFD PBS n=4; HFD DT n=5). Data are presented as mean ± SEM; **p*< 0.05.


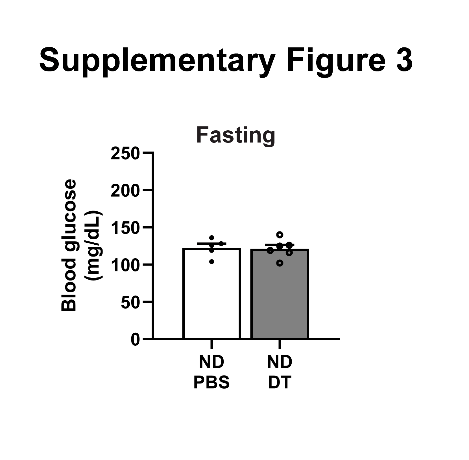


**Figure S3. Inducible depletion of myeloid-derived Zbtb46^+^ cells does not alter glucose homeostasis in lean mice.** Fasting (16 h) blood glucose levels after the inducible depletion challenge (ND PBS n=5; ND DT n=6). Data are presented as mean ± SEM.


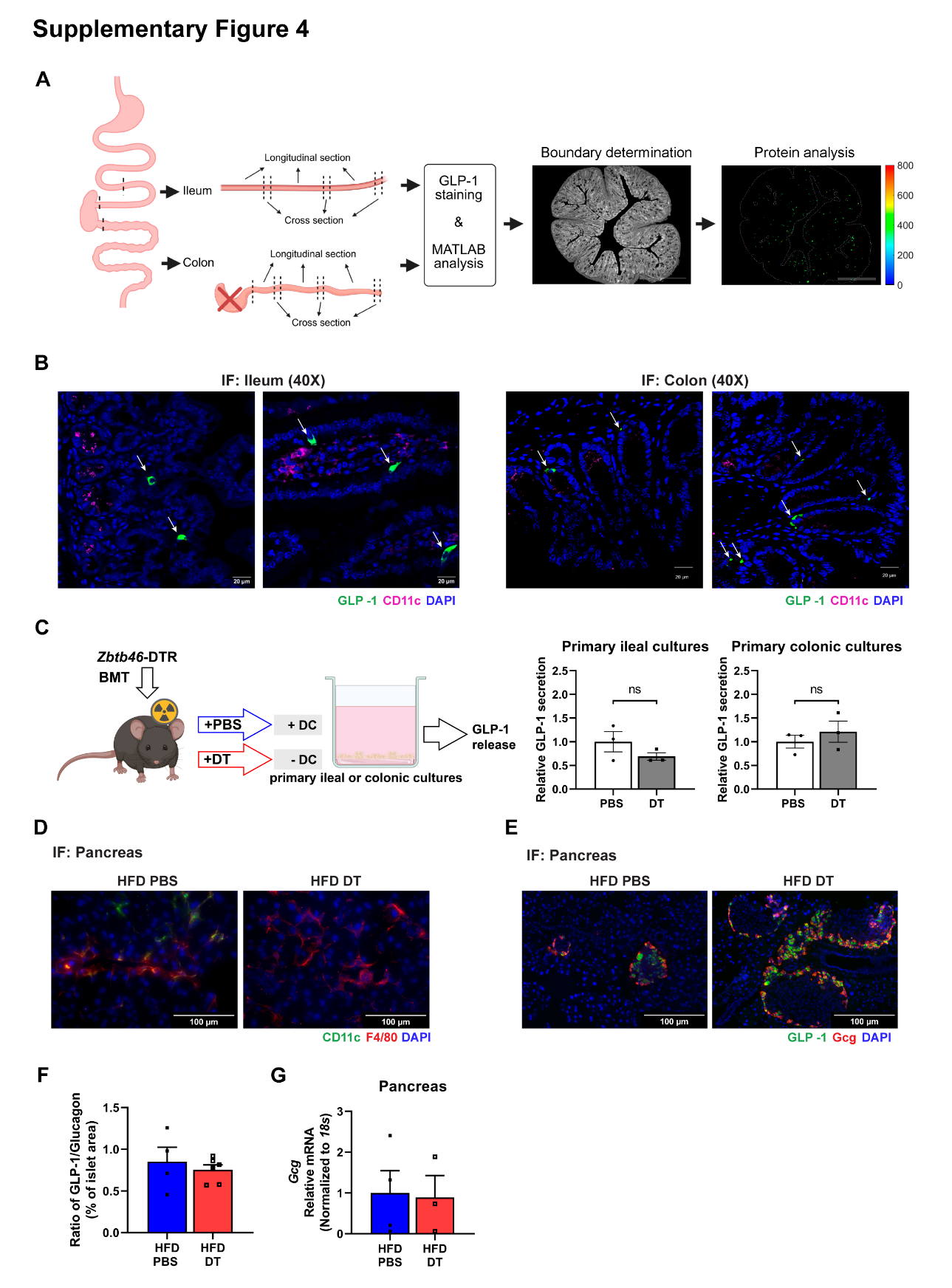


**Figure S4. Inducible depletion of myeloid-derived Zbtb46^+^ cells does not alter local intestinal or pancreatic factors.** (A) Illustration of MATLAB-based analysis of GLP-1 expressions in the distal intestine. Ileum (distal third of small intestine) and colon (from rectum to distal gut) were sectioned into multiple longitudinal and cross sections. At least three of each type were mounted per slide from each mouse and immunofluorescence-stained for GLP-1. Images were captured from each region at 10X or 20X magnifications and analyzed with MATLAB-based scripts. (B) Representatives of immunofluorescence staining of GLP-1^+^ cells (green; white arrow) and CD11c^+^ cells (magenta) in the ileum and colon at 40X magnification. (C) Primary intestinal culture and quantification of GLP-1 secretion from ileum and colon of PBS- or DT-treated *Zbtb46* chimeric mice (n=3 per group). Ileum and colon were digested with Collagenase I/Collagenase XI, and cell suspensions were plated in Matrigel-coated wells plates. After 24-36 h of culture, accumulated GLP-1 release into the media was quantified by ELISA. (D) Representatives of immunofluorescence staining of CD11c^+^ cells (green) and F4/80^+^ cells (red) in pancreas from PBS or DT-treated obese *Zbtb46* chimeric mice. (E-F) Representatives of immunofluorescence staining (E) and its quantification (F) of ratio GLP-1^+^ cells (green) over Glucagon^+^ cells (red) in pancreas from PBS or DT-treated obese *Zbtb46* chimeric mice. One dot represents the average values from individual mouse (HFD PBS n=4; HFD DT n=6). (G) Relative mRNA expression of *Gcg* in pancreas (HFD PBS n=4; HFD DT n=3). Data are presented as mean ± SEM.


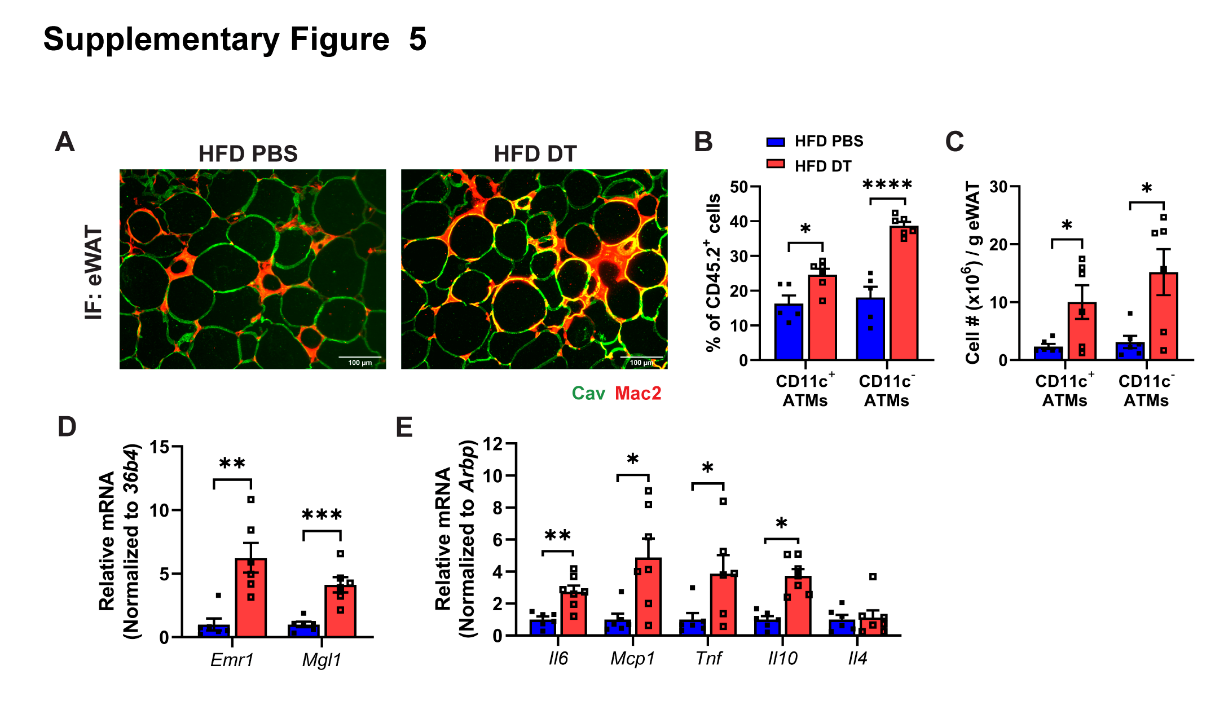


**Figure S5. Inducible depletion of myeloid-derived Zbtb46^+^ cells induces adipose tissue macrophage (ATM) accumulation.** (A) Representatives of immunofluorescence staining of Caveolin^+^ adipocytes (green) and Mac2^+^ ATMs (red) in epididymal white AT (eWAT) from PBS or DT-treated obese *Zbtb46* chimeric mice. (B) Quantification of CD11c^+^ and CD11c^−^ ATMs among leukocytes of donor cells (HFD PBS n=5; HFD DT n=6). (C) Quantification of CD11c^+^ and CD11c^−^ ATMs in cell number per gram (n=6 per group). (D-E) Relative mRNA expression of macrophages signature genes (D) and pro-inflammatory or anti-inflammatory cytokines (E) (HFD PBS n=6; HFD DT n=6-7). Data are represented as mean ± SEM; **p*< 0.05, ** *p*<0.01, *** *p*<0.001, *****p*<0.0001.


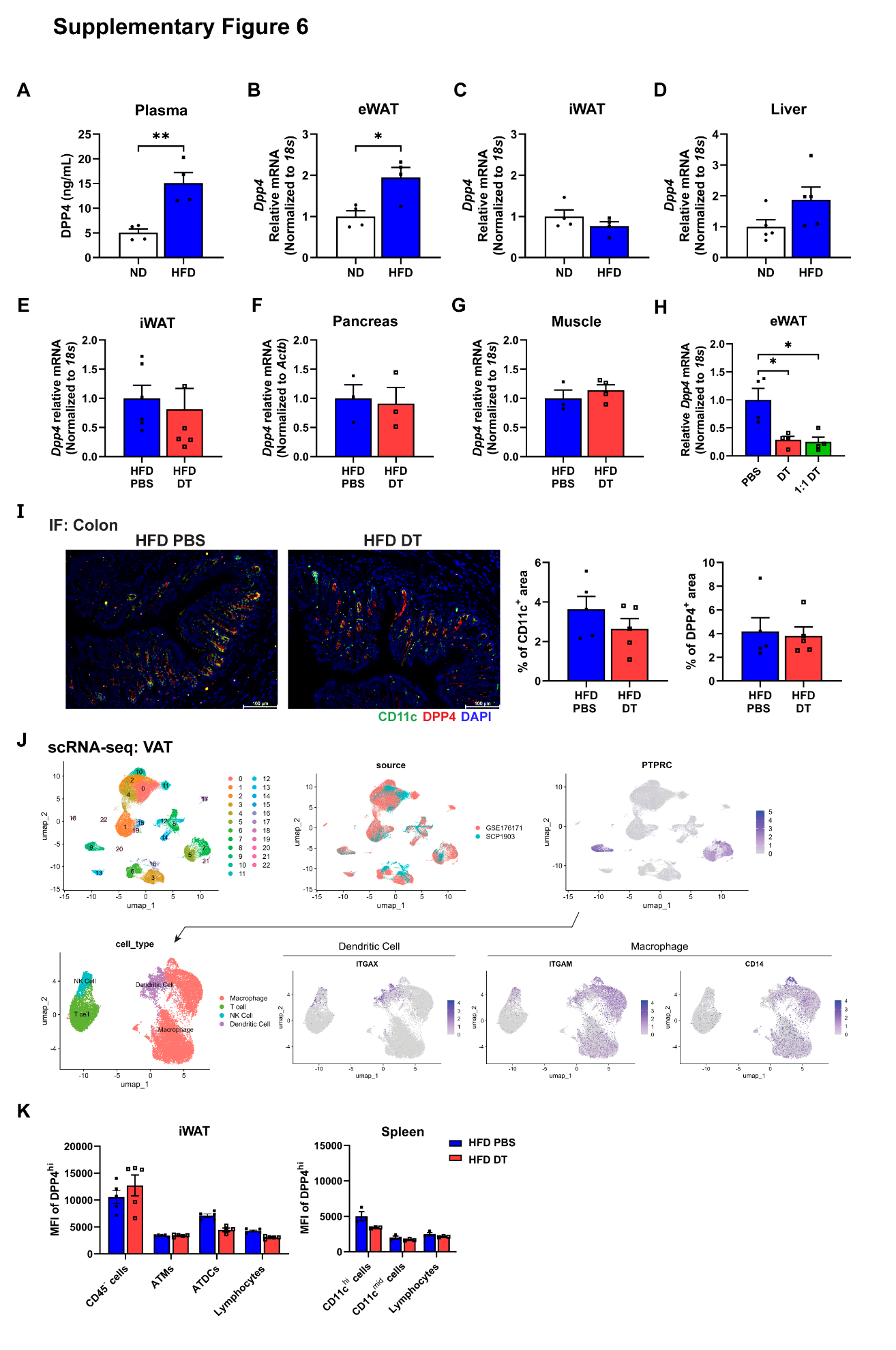


**Figure S6. DPP4 expression in various tissues under lean, obese, and inducible depletion conditions.** (A-D) Diet-induced obesity was used to induce obesity in mice. (A) DPP4 levels in the fasting plasma (n=4 per group). (B-D) Relative mRNA expression of DPP4 in eWAT (B, n=4 per group), iWAT (C, n=4 per group), and liver (D, n=5 per group). (E-I) After inducible depletion challenge in obese mice, relative mRNA expression of *Dpp4* was measured in iWAT (E, HFD PBS n=6; HFD DT n=5), pancreas (F, n=3 per group), and muscle (G, HFD PBS n=3; HFD DT n=4). (H) Relative mRNA expression of *Dpp4* in eWAT from 1:1 DT challenge (n=4 per group). (I) Representatives of immunofluorescence staining and its quantification of CD11c^+^ cells (green) and DPP4^+^ cells (red) in colon from PBS or DT-treated obese *Zbtb46* chimeric mice. Each dot represents the average of measurements from one mouse (n=5 per group). (J) Cluster analysis and immune cell annotation of single-cell RNA sequencing (scRNA-seq) datasets from human VAT. Integrated datasets (GSE176171 and SCP1903) were analyzed using Seurat v5.3.0. Immune cell clusters were defined by PTPRC (CD45), and macrophage and dendritic cell annotated based by canonical markers. (K) Quantification of median fluorescence intensity (MFI) of DPP4^hi^ in iWAT and spleen immune cells from PBS or DT-treated obese *Zbtb46* chimeric mice (iWAT: n=5 per group; spleen: n=3 per group). Data are presented as mean ± SEM; **p*< 0.05, ** *p*<0.01.

**
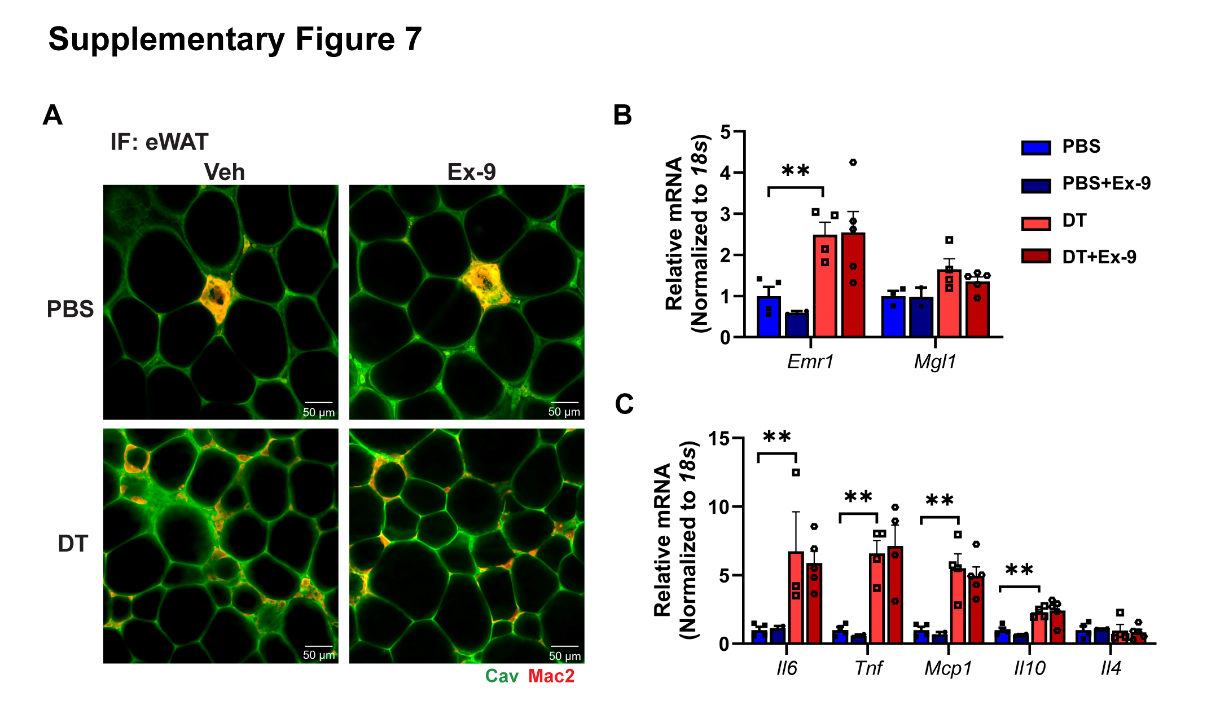
**

**Figure S7. Unaltered profile of epididymal white adipose tissue (eWAT) in obese chimeric *Zbtb46*-DTR mice following Exendin-9 treatment.** (A) Representatives of immunofluorescence staining of Caveolin^+^ adipocytes (green) and Mac2^+^ ATMs (red) in epididymal white AT (eWAT) from Vehicle (veh) or Exendin-9 (Ex-9)-treated PBS or DT obese *Zbtb46* chimeric mice. (B-C) Relative mRNA expression of macrophages signature genes (B) and pro-inflammatory or anti-inflammatory cytokines (C) (PBS n=4; PBS+Ex-9 n=2; DT n=4; DT+Ex-9 n=4-5). Data are presented as mean ± SEM; ** *p*<0.01.

**
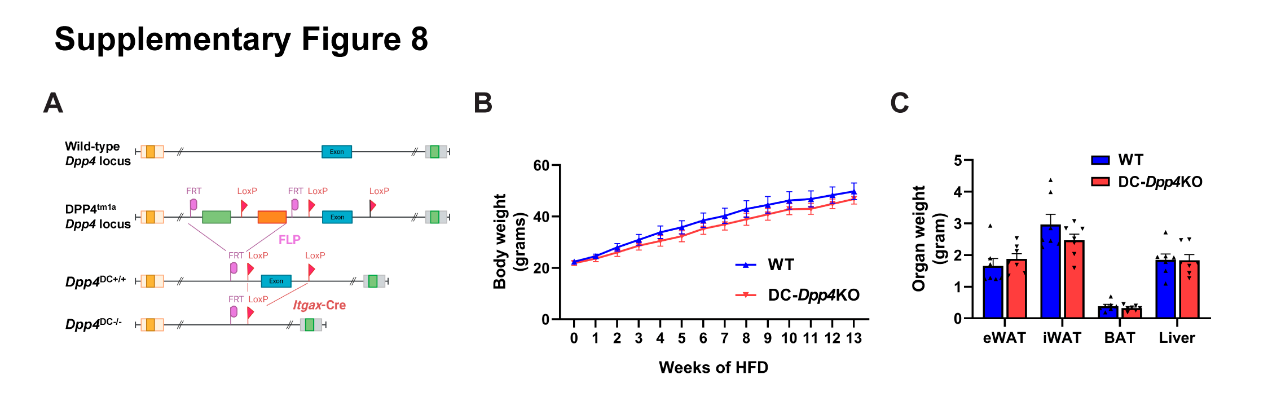
**

**Figure S8. Metabolic profile of DC-*Dpp4*KO mice during HFD challenge.** (A) Schematic of the *Dpp4* locus showing the generation of DC-*Dpp4*KO (*Dpp4*^DC-/-^) mice and their wild-type (WT) littermates (*Dpp4*^DC+/+^). (B) Body weight gain on HFD and (C) final organ weights (n=7 per group). Data are presented as mean ± SEM.

| **Table S1. List of antibodies** | | | | |
| --- | --- | --- | --- | --- |
| **Antibodies** | | | **Source** | **Identifier (#)** |
| *Flow cytometry* | | | | |
| Fixable Viability Dye eFluor® 506 | | | eBioscience | 65-0866-14 |
| FITC anti-mouse CD45.1 Monoclonal Antibody (A20) | | | eBioscience | 11-0453-82 |
| eFluor® 450 anti-mouse CD45.2 Monoclonal Antibody (104) | | | eBioscience | 48-0454-82 |
| PE anti-mouse CD64 (X54-5/7.1) | | | BD Biosciences | BD558455 |
| APC anti-mouse CD11c Monoclonal Antibody (N418) | | | eBioscience | 17-0114-82 |
| PE-Cyanine7 anti-mouse CD11b Monoclonal Antibody (M1/70) | | | eBioscience | 25-0112-82 |
| APC-eFluor® 780 anti-mouse CD3e Monoclonal Antibody (145-2C11) | | | eBioscience | 47-0031-82 |
| PE anti-mouse CD26/DPP4 (H194-112) | | | BioLegend | 137804 |
| PerCP-Cyanine5.5 anti-mouse CD4 Monoclonal Antibody (RM4-5) | | | eBioscience | 45-0042-82 |
| APC anti-mouse CD8a Monoclonal Antibody (53-6.7) | | | eBioscience | 17-0081-82 |
| PE-Cyanine7 anti-mouse FOXP3 Monoclonal Antibody (FJK-16s) | | | eBioscience | 25-5773-82 |
| PE anti-mouse IFN gamma Monoclonal Antibody (XMG1.2) | | | eBioscience | 12-7311-82 |
| APC anti-mouse IL-17A Monoclonal Antibody (eBio17B7) | | | eBioscience | 17-7177-81 |
| Anti-Mouse CD16/CD32 Purified (Fc Block) | | | eBioscience | 14-0161-85 |
|  | | |  |  |
| *Immunoblotting* | | | | |
| Phospho-Akt (Ser473) (D9E) XP(R) Rabbit mAb | | | CST | 4060S |
| Akt | | | CST | 9272S |
| HSP 90α/β | | | Santa Cruz | sc-13119 |
|  | | |  |  |
| *Immunofluorescence* | | | | |
| Mouse Insulin Antibody (2D11-H5) | | | Santa Cruz | sc-8033 |
| Mouse Recombinant Anti-Glucagon antibody (EP3070) | | | Abcam | ab92517 |
| Mouse GLP1 Antibody (4) [Alexa Fluor 488] | | | Novus | NBP2-23558AF488 |
| Mouse CD11c Monoclonal Antibody (N418) | | | eBioscience | 14-0114-82 |
| Mouse F4/80 Monoclonal Antibody (BM8) | | | eBioscience | 14-4801-82 |
| Mouse Caveolin-1 Antibody (7C8) | | | Santa Cruz | sc-53564 |
| Mouse Galectin 3 Monoclonal Antibody (eBioM3/38 (M3/38)) | | | eBioscience | 14-5301-85 |
| Human CD1c/BDCA-1 Antibody (OTI2F4) | | | Abcam | ab156708 |
| Human Recombinant Anti-CD68 antibody [EPR20545] | | | Abcam | ab213363 |
| Human DPPIV/CD26 Antibody | | | Novus | AF1180 |
| **Table S2. List of primers** | | | | |
| **Gene** | **Sequences** | | | |
|  | **Forward** | **Reverse** | | |
| *18s* | TTG ACG GAA GGG CAC CAC CAG | GCA CCA CCA CCC ACG GAA TCG | | |
| *Dpp4* | TAT GCC CAG TTT AAC GAC ACA G | ACA GTT GGA TTC ACA GCT CCT | | |
| *Insr* | CAG TTT GTG GAA CGG TGC TG | CAC ACT TGG TGG GGT CAT CA | | |
| *Irs1* | TCT ACA CCC GAG ACG AAC ACT | TGG GCC TTT GCC CGA TTA TG | | |
| *Irs2* | GAC TTC CTG TCC CAT CAC TTG | TTT CAA CAT GGC GGC GA | | |
| *Emr1* | CCC CAG TGT CCT TAC AGA GTG | GTG CCC AGA GTG GAT GTC T | | |
| *Mgl1* | TGA GAA AGG CTT TAA GAA CTG GG | GAC CAC CTG TAG TGA TGT GGG | | |
| *Il-6* | TAG TCC TTC CTA CCC CAA TTT CC | TTG GTC CTT AGC CAC TCC TTC | | |
| *Mcp-1/Ccl2* | TTA AAA ACC TGG ATC GGA ACC AA | GCA TTA GCT TCA GAT TTA CGG GT | | |
| *Tnf* | CCC TCA CAC TCA GAT CAT CTT CT | GCT ACG ACG TGG GCT ACA G | | |
| *Il12b (p40)* | CGA GAC TCT GAG CCA CTC AC | CGT GAA CCG TCC GGA GTA AT | | |
| *Ifng* | ATG AAC GCT ACA CAC TGC ATC | CCA TCC TTT TGC CAG TTC CTC | | |
| *Il10* | GCTCTTACTGACTGGCATGAG | CGCAGCTCTAGGAGCATGTG | | |
| *Il4* | GGT CTC AAC CCC CAG CTA GT | GCC GAT GAT CTC TCT CAA GTG AT | | |
| *Zbtb46* | ATC ACT TCT CAC TAC CGG CAT | ACG TTC TTA TGT GCC TTG AAG AC | | |
| *H2-Ab1* | CAC TCT GGT CTG TTC GGT GAC | CCT CTC CCT GAT GAG GGG TC | | |
| *Ccr7* | TCA TTG CCG TGG TGG TAG TCT TCA | ATG TTG AGC TGC TTG CTG GTT TCG | | |
| *Btla* | GCC ATT CAG TAA CCA TCC ATG TG | GCA AGG TGT AAA GCA GCC AAG TC | | |
| *Cd80* | ACC CCC AAC ATA ACT GAG TCT | TTC CAA CCA AGA GAA GCG AGG | | |
| *Cd86* | TGT TTC CGT GGA GAC GCA AG | TTG AGC CTT TGT AAA TGG GCA | | |
| *Gcg* | ACA CCA AGA GGA ACC GGA AC | TCT TCT GGG AAG TCT CGC CT | | |
